# Challenging ChatGPT 3.5 in Senology—An Assessment of Concordance with Breast Cancer Tumor Board Decision Making

**DOI:** 10.3390/jpm13101502

**Published:** 2023-10-16

**Authors:** Sebastian Griewing, Niklas Gremke, Uwe Wagner, Michael Lingenfelder, Sebastian Kuhn, Jelena Boekhoff

**Affiliations:** 1 Institute for Digital Medicine, University Hospital Marburg, Philipps-University Marburg, Baldingerstraße, 35043 Marburg, Germany; sebastian.kuhn@uni-marburg.de; 2 Department of Gynecology and Obstetrics, University Hospital Marburg, Philipps-University Marburg, Baldingerstraße, 35043 Marburg, Germany; gremken@staff.uni-marburg.de (N.G.); uwe.wagner@uk-gm.de (U.W.); jboekhof@med.uni-marburg.de (J.B.); 3 Institute for Healthcare Management, Chair of General Business Administration, Philipps-University Marburg, Universitätsstraße 24, 35037 Marburg, Germany; lingenfe@wiwi.uni-marburg.de

**Keywords:** artificial intelligence, large language models, gynecology, oncology, tumor board

## Abstract

With the recent diffusion of access to publicly available large language models (LLMs), common interest in generative artificial-intelligence-based applications for medical purposes has skyrocketed. The increased use of these models by tech-savvy patients for personal health issues calls for a scientific evaluation of whether LLMs provide a satisfactory level of accuracy for treatment decisions. This observational study compares the concordance of treatment recommendations from the popular LLM ChatGPT 3.5 with those of a multidisciplinary tumor board for breast cancer (MTB). The study design builds on previous findings by combining an extended input model with patient profiles reflecting patho- and immunomorphological diversity of primary breast cancer, including primary metastasis and precancerous tumor stages. Overall concordance between the LLM and MTB is reached for half of the patient profiles, including precancerous lesions. In the assessment of invasive breast cancer profiles, the concordance amounts to 58.8%. Nevertheless, as the LLM makes considerably fraudulent decisions at times, we do not identify the current development status of publicly available LLMs to be adequate as a support tool for tumor boards. Gynecological oncologists should familiarize themselves with the capabilities of LLMs in order to understand and utilize their potential while keeping in mind potential risks and limitations.

## 1. Introduction

Medical research increasingly explores the application of artificial intelligence (AI) and novel machine learning methods that adaptively and automatically process heterogeneous health data to enable personalized medical treatment [1]. In light of modern health challenges, including the COVID-19 pandemic, deep and machine learning methods have been proven to facilitate medical decision making and provide benefits to patients and caregivers beyond the previously known non-medical areas of application of the technology [2,3,4,5,6]. Particularly for the diagnosis, treatment and follow-up of highly complex and chronic diseases, as is the case in oncology, there is growing interest in corresponding clinical applications of individualized precision medicine [7,8]. In view of the demographic development and rapid aging of the population in central Europe, a continuing increase in oncological disease is predicted [9]. In addition, methodological innovations such as patient-specific genomic sequencing are becoming accessible and cost-effective [10]. This leads to an almost exponential increase in oncology treatment data and medical knowledge through novel research opportunities [11]. While this treasure trove of oncological health data opens up a new dimension of scientific possibilities, it is beyond the capabilities of human cognitive processing and calls for the application of automated data computing [12,13].

Professionally trained clinical decision support systems (CDSSs), i.e., CancerLinq, OncoDoc or IBM Watson for Oncology, have proven their capability to process these data in large-scale retrospective, observational studies [14,15]. Nevertheless, the recent diffusion of access to public large language models (LLMs) takes the handling of AI-based applications for medical purposes to a new level. Since generative AI-based LLM ChatGPT was made available to the general public by OpenAI (San Francisco, CA, USA) in November 2022, the exploration of the collaboration between human cognition and intelligent machines has rapidly gained public interest. Swiftly, generative AI and LLMs have made their way into our daily lives, not stopping at how we manage our own health [16]. After just one year, questioning of ChatGPT’s about personal health issues has become a normality for technology-savvy patients.

Initial pilot studies indicate acceptable accuracy of LLMs in clinical decision making and general medical knowledge throughout the clinical workflow [17]. With regard to breast cancer care, Rao et al. were able to provide evidence of the application of ChatGPT for radiology decision making and screening purposes, justifying its responsible use for radiology services [18]. The available studies argue for the evaluation of further use cases and greater accuracy before the implementation of LLMs in the clinical treatment process [18]. With respect to oncological treatment, research is exploring the consistency of publicly available LLMs and has intensified the discussion about the question whether AI-assisted decision making will change the way tumor boards are conducted [19,20,21]. In gyne-oncology, only two studies have investigated the performance of publicly available LLMs in breast cancer tumor board decision making [22,23]. While the authors advocate for the promising potential of LLMs in breast cancer tumor boards and clinical oncology, the scientific approach to handling the new technology is still in its infancy. Lukac et al. and Sorin et al. limited their study populations to a small number of randomly selected patient profiles; used a short input model that does not do justice to the information contained in the actual tumor board presentation; partially excluded high-complexity cases, i.e., primary distant metastasis; or neglected to distinguish between different breast cancer treatment options [22,23].

This explorative pilot study aims to extend the results reported by Lukac et al. and Sorin et al. to evaluate the concordance of treatment decisions made by the most prominent publicly available LLM, ChatGPT 3.5 by Open AI, with those of the multidisciplinary tumor board (MTB) of a gynecological oncology center in Germany. The study design is therefore based on patient profiles reflecting the patho- and immunomorphological diversity of primary breast cancer, including primary metastasis and precancerous tumor stages, and extends to a detailed and structured input model. In addition, the entire bandwidth of treatment options for breast cancer, including surgical re-excision, endocrine, chemotherapy, radiation therapy and genetic testing, is evaluated separately.

## 2. Patients and Methods

### 2.1. Patient Profiles

To capture the patho- and immunomorphological diversity of breast cancer in comprehensive manner, 20 patient profiles were designed by the head of the investigated gynecologic oncology center in orientation to the current immunohistochemical and molecular subtypes in accordance with the current breast cancer guidelines of the German Association of Gynecology and Obstetrics (DGGG) [24]. In addition, a differentiation by nodal status and postmenopausal status was performed for each subtype (P1–P20, as shown in Table 1).

**Figure 1:**
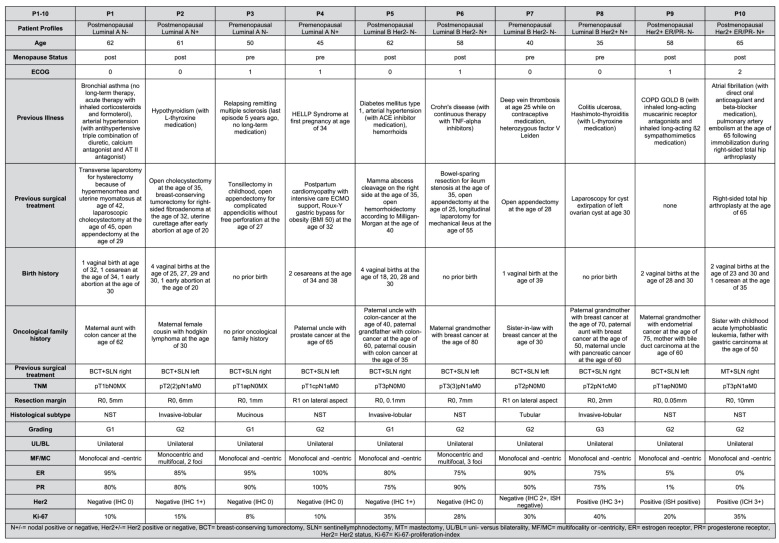
Detailed patient profiles (P1–P10).

**Figure 2:**
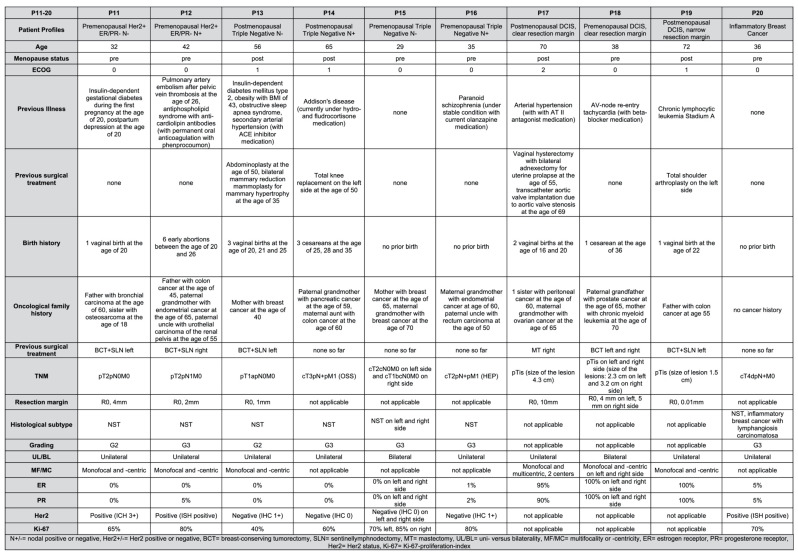
Detailed patient profiles (P11–P20).

Subsequently, the patient profiles were completed to include patient age, ECOG (Eastern Cooperative Oncology Group Performance Scale), previous illness, previous surgical treatment, birth history and oncological family history (as shown in Figure 1 and Figure 2). Further diagnostic data were designed to the extent of pTNM classification, minimal resection margin (R0/R1, in mm), histological classification (non-special-type NST, invasive lobular, tubular or mucinous), grading (according to Bloom–Richardson–Elston score [25]), unilaterality versus bilaterality, and multifocality or -centricity. The data with regard to immunohistochemical and molecular subtyping were determined to the extent of hormonal status (estrogen receptor (ER), 0–100%; progesterone receptor (PR), 0–100%), Her2 status (immunohistology (IHC) or in situ hybridization (ISH) and Ki-67 proliferation index (0–100%). For data security and compliance reasons, the profiles are fictitious and do not reflect actual patient cases. Based on this, we notified the university’s ethics committee and were informed that ethical approval is not required.

### 2.2. Extended Input Model

**Figure 3:**
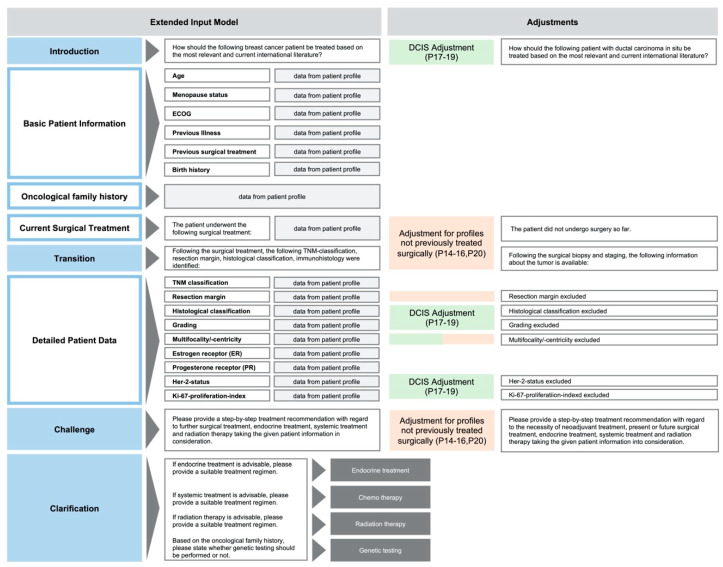
Extended input model.

The following extended input model was applied based on the aforementioned data from each patient profile. The structuring includes an introductory sentence, followed by basic profile-specific health data and the formulation of an oncological family history. Furthermore, the current surgical treatment of the tumor is stated, leading to a transition to detailed data about the lesion’s patho- and immunomorphological characteristics. Lastly, the specific task (or challenge) is presented in connection with a clarification about the advisable treatment options (as shown in Figure 3).

Wording was slightly adjusted for patient profiles not previously treated surgically (P14–P16 and P20) and for cases of ductal carcinoma in situ (DCIS) (P17–P19).

### 2.3. Model Execution

Prior to model execution, a randomization of the profile sequence was executed (see File S1). Furthermore, a blinded version of the standardized input model without reference to the patient profile number was created. Afterwards, model execution was performed on 21 July 2023 by presenting one profile after another to the publicly available ChatGPT 3.5 (OpenAI LP, San Francisco, CA, USA). The study design focuses on testing the ChatGPT 3.5, as it is publicly available at no charge and, thus, primarily used by patients and healthcare professionals in a medical context at the present time. Correspondingly, the blinded version of the input model was translated to German using DeepL AI-based translation services (DeepL SE, Cologne, Germany), and the predefined patient profiles were discussed in the same randomized order by the actual multidisciplinary tumor board (MTB) of the investigated gynecologic oncology center on the same date. MTB participants were informed about the execution of an experiment without any information about the study design, and they were given the option to decline participation. Accordingly, they were instructed to treat patient cases and determine treatment decisions as they would in the regular course of tumor board decision making. On the specific date, the tumor board consisted of four specialized gyne-oncologists, two gynecologists, two oncologists, one human geneticist, one radiation physician, one pathologist and two gynecological residents. The head of the gynecologic oncology center under study did not participate in the study due to knowledge of the patient profiles.

### 2.4. Concordance Assessment

As specified in the input model, recommendations of the LLM and MTB were analyzed with respect to the treatment options of surgical treatment (ST), endocrine treatment (ET), systemic treatment or chemotherapy (CT), radiation therapy (RT) and genetic testing (GT). As such, they were measured in a bivariate manner (treatment option recommended = yes; not recommended = no). Concordance assessment of LLM and MTB treatment was performed in terms of descriptive statistical evaluation (in %) for each individual patient profile and for each subordinate treatment option separately. As LLMs are designed to generate a relative formulation, formulation of possible treatment was rated as recommended treatment.

## 3. Results

### 3.1. Treatment Recommendation Frequency

In total, 61 treatment recommendations were proposed by the LLM, and 48 were proposed by the MTB for the predefined patient profiles. The greatest difference in recommendation frequency results was obtained for GT (as shown in Table 2).

### 3.2. Concordance Assessment Per Patient Profile

Concordance between LLM and MTB recommendations was registered for half of the patient profiles (CC_Total_ = 50.0%; 10 of 20 PP). Overall concordance for invasive breast cancer patients (CC_BreastCancer_), excluding DCIS profiles P17 to 19 amounted to 58.8% (10 of 17 PP). Removing GT from the assessment resulted in full concordance (CC_Total_NoGT_) for 68.4% (13 of 19 PP) of all PP and 81.25% (13 of 16 PP) for invasive breast cancer PP (CC_BreastCancer_NoGT_). PP 7 had to be excluded from the partial evaluation because the MTB recommended further testing using Endopredict^®^ (Myriad Service GmbH, Munich, Germany) to assess the need for chemotherapy for the specific patient profile.

### 3.3. Concordance Assessment Per Treatment Option

The MTB recommended surgical re-excision (ST) for three, in comparison to two PP in the case of the LLM. Concordance for ET, CT, RT and GT amounted to CC_ET_ = 75.0% (15 of 20 PP), CC_CT_ = 94.5% (18 of 19 PP), CC_RT_ = 95.0% (19 of 20 PP) and CC_GT_ = 70.0% (14 of 20 PP) (as shown in Table 3). With regard to CT, PP 7 had to be removed from the assessment based on the aforementioned MTB decision on further breast cancer prognostic testing.

### 3.4. Comparative Results of LLM and MTB Treatment Decisions

A direct comparison between the treatment recommendations of the LLM and MTB is presented in Table 4. Further details regarding qualitative treatment recommendations (i.e., aromatase inhibitor versus tamoxifen treatment in ET or specific chemotherapy regimen) are included in File S1.

## 4. Discussion

### 4.1. Main Findings

This observational study shows that ChatGPT 3.5, a publicly available LLM, can provide treatment recommendations for breast cancer patients that are consistent with multidisciplinary tumor board decision making of a gynecologic oncology center in Germany. This observation is important, as it adds to previous findings by applying an extended standardized input model, assessing a broader spectrum of patho- and immunomorphological breast cancer subtypes, including primary metastatic and precancerous tumor stages, in a structured manner, in addition to evaluating possible breast cancer treatment options separately. With CC_Total_ and CC_BreastCancer_ amounting to 50.0% and 58.8%, respectively, the general level of concordance observed in this study lies in the middle of that reported in preceding studies by Lukac et al. and Sorin et al. The authors of these studies showed that the congruence of the chatbot’s recommendations with those of the specific tumor board amounted to 70% (Sorin et. al.) and 16.05% (Lukac et al.) [22,23]. Once retrieving the GT option from assessment, as the necessity of genetic testing has not previously been measured equivalently by the colleagues, the study provides a total concordance level that matches the findings of Sorin et al. (CC_Total_NoGT_ = 68.4%). Furthermore, this level of accuracy meets the average performance of ChatGPT of 71.8% as measured by Rao et al. in their first-of-its-kind study that assessed the AI tool’s potential use along the entire clinical workflow, including diagnostic workup, diagnosis and clinical management [17]. While Sorin et al. refrained from further distinguishment between treatment options, Lukac et al. did so without evaluating the concordance between these subgroups. Thus, this study adds to these previous findings by showing that concordance for individual treatment options, including ET, CT and RT (CC_ET_ = 75.0%, CC_CT_ = 94.5%, CC_RT_ = 95.0%), stands out considerably. However, compared to a professionally trained CDSS, i.e., Watson for Oncology, which has been proven to achieve overall concordance of up to 93% for breast cancer cases, we rate the LLM’s performance as rather low [14,15].

### 4.2. Further Findings

#### 4.2.1. Garbage in–Garbage Out

By applying an extended input model with detailed patient profiles (see Figure 1, Figure 2 and Figure 3), this study demonstrates that the chatbot can only perform to the level of quality of the data it is fed. As such, it follows the principle of “garbage in–garbage out” for AI-enabled precision medicine applications [17,26]. While Lukac et al. argue that the chatbot does neglect neoadjuvant treatment, our extended input model contradicts this finding [23]. Once explicably asked to consider neoadjuvant treatment, ChatGPT 3.5 successfully identifies suitable situations for neoadjuvant treatment and provides detailed explanation, even mentioning a suitable chemotherapy regimen. Furthermore, our colleagues argue that the LLM does not include current or ongoing studies, which is based on the fact that ChatGPT 3.5 is limited to data published until September 2021. Thus, the LLM is not able to learn the latest science on oncology issues, so it needs to be trained on the latest standards in order to not fall back in “garbage out” situations. In the other hand, medical laypersons will have a hard time recognizing appropriate situations compared to oncology experts. In order not to fall into corresponding “garbage in–garbage out” situations, professionally trained CDSSs receive previously filtered high-quality data and literature as input for its computing process [14,15].

#### 4.2.2. Lack of Consistency in Health Data Use

Although the study design presents an extended input model with a larger amount of detailed health data to the LLM, we must confirm the finding of our colleagues that ChatGPT partially fails to successfully and consistently take individual patient information into account. Thus, Lukac et al. stated that the LLM did not take age into consideration for systemic treatment in elderly patients [23]. Beyond that, extended input model applied herein provides the LLM with a detailed patient history on ECOG, previous illness, surgical history and birth history. Nevertheless, the chatbot did not apply this important background information to back up treatment decisions.

In contrast to this, the LLM successfully accessed the majority of the further provided health data, i.e., age and pre- or postmenopausal status were used to distinguish between aromatase inhibitor and selective estrogen receptor modulators or ovarian suppression by GnRH agonists, which confirms the findings of Sorin et al. and Lukac et al. [22,23]. As the extended input model explicitly asked for a suitable treatment regimen, the chatbot did provide correct medication (i.e., 2.5 mg letrozole p.o. daily) and treatment duration for some patient profiles. Novel findings of this study include the surgical treatment and minimal resection margin being commented on in terms of correctness and sufficiency, the necessity of re-excision being recognized for R1 situations and bilaterality being identified with successful distinguishment between left and right side. With regard to immunohistochemical and molecular subtypes, the LLM successfully took hormonal status, grading, Her2 status and Ki-67 proliferation index into account for treatment planning. Thus, it identified triple-negative cancer types; distinguished between Her2-positive and -negative situations, which resulted in the recommendation of targeted therapies (i.e., trastuzumab); and recognized primary metastatic situations. Furthermore, by providing an oncological family history for each patient profile, decision making with regard to genetic testing was tested to a novel extent. While Lukac et al. only acknowledged the LLM’s potential to recognize the possibility of hereditary risk in a young patient with advanced breast cancer, this study’s findings expand on this finding by showing its capacity to successfully interpret oncological family histories. Thus, the chatbot not only identifies a specific profile being prone to hereditary breast and ovarian cancer (HBOC) but also makes a differentiation for profiles with colorectal or endometrial disease, drawing a link to Lynch syndrome (i.e., P16 or P5).

By providing the extended health data to the LLM and explicitly requesting a suitable regimen for possible endocrine, radiation and chemotherapy treatment, the chatbot provided individualized treatment decisions for patient profiles in connection with a structured and detailed explanation. Furthermore, by confronting the LLM with diverse patient profiles, including high-complexity cases with primary metastasis, it showed potential to cover broader patho- and immunomorphological diversity of breast cancer in comparison to previous studies. Nevertheless, this study points out a lack of consistency in terms of when and how the LLM used the specific data.

#### 4.2.3. Stepping into the Trip Trap

Another crucial limitation of the LLM becomes evident as it steps into predefined trip traps, resulting in raw treatment mistakes, which the MTB easily evaded. The chatbot recommended genetic testing based on a sister-in-law with breast cancer history (P7), stating the necessity of testing for BRCA 1 and 2 mutations. Furthermore, it neglected the necessity of re-excision for DCIS with a narrow resection margin of 0.01 mm (P19). Such fraudulent decisions hold the potential to adversely affect treatment decisions and negatively impact the patient’s health situation. This confirms a critical challenge of natural language models in the context of breast cancer decision making. Regarding the notion of misalignment and hallucination, research recognizes a major challenge for LLMs, which tend to hallucinate unintended text, limiting their current level of development for use in real-world scenarios [27]. As the sister-in-law example shows, the stochastic nature of LLMs can be quickly exploited by misaligning simple designed inputs, resulting in fraudulent responses [28]. Although the performance of LLMs appears impressive when assessed superficially, it proves to be prone to misinterpretation and hallucinations despite being equipped with sufficient information, which limits its application in the medical context [17]. Even small errors in judgment can lead to significant treatment errors for breast cancer that pose a negative risk to a patient’s health. The difference between 61 treatment recommendations from the LLM and the 48 from the MTB underlines the LLM’s over-recommendation tendency, which ultimately may lead to overtreatment and lack of individualized treatment decision making, i.e., the chatbot recommended endocrine treatment for all DCIS profiles (P17-P19), as well as situations with low ER and PR positivity (P16 and P9), for invasive breast cancer, which are can-do decisions but not necessarily must-do. As one of the main motives of AI use is based on the adaptive automatic processing of heterogeneous health data to enable personalized medical treatment decisions, the current state of publicly available LLMs does not live up to this expectation [1].

### 4.3. Limitations and Suggestions for the Future

We acknowledge that this manuscript represents a pilot study that explores a novel scientific approach to the application of publicly available LLM ChatGPT 3.5 in the context of breast cancer care. Owing to the nature of explorative, small-scale pilot studies, the current study design includes a considerable number of limitations.

The present study design follows a single-center approach, which tests the LLM’s performance against the decision making of a singular certified gynecologic oncology center in Germany. In order to enable the transferability and generalizability of the results, an extension to a multicenter and -national evaluation would be desirable. As such, the decisions of the investigated MTB are based on German standards according to the German Society of Gynecology and Obstetrics guidelines and may differ in an international comparison. Furthermore, this explorative study contains a limited number of patient profiles. Coherent to the testing of CDSS accuracy, the evaluation of LLMs should be extended to large-scale observational studies to allow for the assessment of statistical significance. This work is limited to testing of ChatGPT version 3.5. Follow-up studies should extend the study design to a comparability assessment by including the not-yet-freely available ChatGPT 4 or other LLMs. It remains important to note that due to the nature of a large language models, this observational study is limited to the qualitative interpretation of the LLM’s wording and argumentation.

Nevertheless, the study design expands on preceding scientific work through the application of an extended input model and taking a broader spectrum of the patho- and immunohistological variety and treatment diversity of breast cancer care into consideration. Beyond that, we consider the scientific evaluation of publicly available LLMs to be necessary. Due to their diffusion of use in society, they are and will inevitably be used in medical contexts. At present, the scientific use and clinical application of LLMs are still in their infancy. This study has added to the specific setting of tumor board decision making. Nevertheless, the evaluation of further use cases throughout the clinical process are necessary to identify situations in which LLMs can create the greatest impact and benefits for patients and healthcare providers. We recognize that the qualities of LLMs will lead to them occupying a place in clinical medicine, but the optimal area of integration has yet to be defined.

## 5. Conclusions

As this pilot study explores the application of LLMs in oncological decision making, we also confronted the chatbot with the question of why it should, in perspective, be integrated in breast cancer tumor boards:

**Authors:** “Please provide a short argumentation of 100 words on why the use of ChatGPT should, in perspective, be integrated in breast cancer tumor boards?”.
**ChatGPT 3.5:** “Integrating ChatGPT into breast cancer tumor boards offers invaluable benefits. Firstly, it ensures access to the latest medical knowledge, keeping oncologists up-to-date with rapidly evolving treatments and research. Secondly, ChatGPT can assist in analyzing complex patient data, improving diagnostic accuracy and treatment planning. Its ability to sift through vast medical literature and patient records swiftly aids in personalized care. Furthermore, it enhances communication within multidisciplinary teams, streamlining information sharing and decision-making. Overall, ChatGPT’s integration empowers healthcare professionals with cutting-edge insights, potentially leading to more informed, efficient, and effective breast cancer care”.

Technological advancement and further development of artificial intelligence (AI) and novel machine learning (ML) methods are projected to disrupt medical care and research by opening up a new dimension of personalized medical treatment decision making [8,11]. The abundance of health data driven by the rapid increase in medical knowledge and modern technological possibilities requires research-driven collaboration with human cognition and automated data processing. Furthermore, the ongoing diffusion of access and user acceptance of publicly available AI tools, including LLMs like ChatGPT, call for scientific monitoring of how to handle their application in medical care and research [26,29]. Few international studies have assessed the accuracy of LLMs for oncological decision making in comparison to MTBs. Although the technological readiness of public LLMs does not meet the level of accuracy required for individualized care decisions for breast cancer, previous studies have advocated for their potential as support tools for breast cancer tumor boards [23,30]. By challenging LLM ChatGPT 3.5 with an extended input model and detailed health data, this study adds to preceding findings and confirms the partial concordance of LLM and MTB decision making for a broader spectrum of care situations for breast cancer. Nevertheless, as the LLM makes considerably fraudulent decisions, which hold the potential to adversely affect treatment decisions and negatively impact the patient’s health situation, we do not identify the current development status of publicly available LLMs to be adequate as support tools for tumor boards. Neither does the chatbot fulfill its own formulated qualities. In contrast, we reserve this area of high complexity and individualized treatment planning for oncological experts with, in perspective, increased support from professionally trained CDSSs [14,15]. Nevertheless, we acknowledge that LLMs will have a place in clinical medicine. Due to their explanatory power, they are powerful tools that can support patients along their care journey; inform and educate patients about their personal cancer diagnosis; facilitate physicians’ access to relevant information by enhancing their up-to-date knowledge; and automate routine medical routine, i.e., automation of discharge summaries [17,22,30,31]. Gynecological oncologists should familiarize themselves with the capabilities of LLMs in order to understand and utilize their potential while keeping in mind potential risks and limitations.

## Figures and Tables

**Table 1:** Generic patient profiles (P1–P20).

Patient Profiles				
Immunohistochemical and Molecular Subtype	Postmenopausal		Premenopausal	
	Nodal Negative	Nodal Positive	Nodal Negative	Nodal Positive
Luminal A	P1	P2	P3	P4
Luminal B	P5	P6	P7	P8
Her2 positive	P9	P10	P11	P12
Triple negative	P13	P14	P15	P16
DCIS	P17		P18	
DCIS with narrow resection margin	P19			
Inflammatory breast cancer				P20

**Table 2:** Treatment recommendation frequency.

Treatment Option	ST		ET		CT		RT		GT	
Model Execution	LLM	MTB	LLM	MTB	LLM	MTB	LLM	MTB	LLM	MTB
Recommendation frequency	2	3	13	8	13	11	16	15	17	11

ST = surgical treatment; ET = endocrine treatment; CT = chemotherapy; RT = radiation therapy; GT = genetic testing.

**Table 3:** Concordance assessment.

PP		ST	ET	CT	RT	GT	CC per PP
Postmenopausal Luminal A N−	1	yes	yes	yes	yes	no	no
Postmenopausal Luminal A N+	2	yes	yes	no	yes	no	no
Premenopausal Luminal A N−	3	yes	yes	yes	yes	yes	yes
Premenopausal Luminal A N+	4	yes	yes	yes	yes	yes	yes
Postmenopausal Luminal B Her2− N−	5	yes	yes	yes	yes	yes	yes
Postmenopausal Luminal B Her2− N+	6	yes	yes	yes	yes	no	no
Premenopausal Luminal B Her2− N−	7	yes	yes	n.a.	yes	no	no
Premenopausal Luminal B Her2+ N+	8	yes	yes	yes	yes	yes	yes
Postmenopausal Her2+ ER/PR− N−	9	yes	no	yes	yes	no	no
Postmenopausal Her2+ ER/PR− N+	10	yes	yes	yes	yes	no	no
Premenopausal Her2+ ER/PR− N-	11	yes	yes	yes	yes	yes	yes
Premenopausal Her2+ ER/PR− N+	12	yes	yes	yes	yes	yes	yes
Postmenopausal Triple Negative N−	13	yes	yes	yes	yes	yes	yes
Postmenopausal Triple Negative N+	14	yes	yes	yes	yes	yes	yes
Premenopausal Triple Negative N−	15	yes	yes	yes	yes	yes	yes
Premenopausal Triple Negative N+	16	yes	no	yes	yes	yes	no
Postmenopausal DCIS, clear resection margin	17	yes	no	yes	no	yes	no
Premenopausal DCIS, clear resection margin	18	yes	no	yes	yes	yes	no
Postmenopausal DCIS, narrow resection margin	19	no	no	yes	yes	yes	no
Inflammatory Breast Cancer	20	yes	yes	yes	yes	yes	yes
	**CC per TO**	**95.0%**	**75.0%**	**94.7%**	**95.0%**	**70.0%**	**50.0%**

PP = patient profiles; yes = concordance between LLM and MTB; no = no concordance between LLM and MTB; PP = patient profile; ST = surgical treatment; ET = endocrine treatment; CT = chemotherapy; RT = radiation therapy; GT = genetic testing; CC per PP = concordance per patient profile; CC per TO = concordance per treatment option; N+/− = nodal positive or negative; Her2+/− = Her2 positive or negative; n.a. = not applicable.

**Table 4:** Comparative results.

PP		ST		ET		CT		RT		GT	
		LLM	MTB	LLM	MTB	LLM	MTB	LLM	MTB	LLM	MTB
Postmenopausal Luminal A N−	1	no	no	yes	yes	no	no	yes	yes	yes	no
Postmenopausal Luminal A N+	2	no	no	yes	yes	yes	no	yes	yes	yes	no
Premenopausal Luminal A N−	3	no	no	yes	yes	no	no	yes	yes	no	no
Premenopausal Luminal A N+	4	yes	yes	yes	yes	no	no	yes	yes	no	no
Postmenopausal Luminal B Her2− N−	5	no	no	yes	yes	no	no	yes	yes	yes	yes
Postmenopausal Luminal B Her2− N+	6	no	no	yes	yes	yes	yes	yes	yes	yes	no
Premenopausal Luminal B Her2− N−	7	yes	yes	yes	yes	yes	n.a.	yes	yes	yes	no
Premenopausal Luminal B Her2+ N+	8	no	no	yes	yes	yes	yes	yes	yes	yes	yes
Postmenopausal Her2+ ER/PR− N−	9	no	no	yes	no	yes	yes	yes	yes	yes	no
Postmenopausal Her2+ ER/PR− N+	10	no	no	no	no	yes	yes	yes	yes	yes	no
Premenopausal Her2+ ER/PR− N−	11	no	no	no	no	yes	yes	yes	yes	yes	yes
Premenopausal Her2+ ER/PR− N+	12	no	no	no	no	yes	yes	yes	yes	yes	yes
Postmenopausal Triple Negative N−	13	no	no	no	no	yes	yes	yes	yes	yes	yes
Postmenopausal Triple Negative N+	14	no	no	no	no	yes	yes	no	no	yes	yes
Premenopausal Triple Negative N−	15	no	no	no	no	yes	yes	no	no	yes	yes
Premenopausal Triple Negative N+	16	no	no	yes	no	yes	yes	no	no	yes	yes
Postmenopausal DCIS, clear resection margin	17	no	no	yes	no	no	no	yes	no	yes	yes
Premenopausal DCIS, clear resection margin	18	no	no	yes	no	no	no	yes	yes	yes	yes
Postmenopausal DCIS, narrow resection margin	19	no	yes	yes	no	no	no	yes	yes	no	no
Inflammatory Breast Cancer	20	no	no	no	no	yes	yes	no	no	yes	yes

PP = patient profiles; yes = treatment recommended; no = treatment not recommended; PP = patient profile; ST = surgical treatment; ET = endocrine treatment; CT = chemotherapy; RT = radiation therapy; GT = genetic testing; LLM = large language model; MTB = multidisciplinary tumor board.

## Data Availability

The data presented in this study are available in File S1.

## Supplementary Materials

The following supporting information can be downloaded at: https://www.mdpi.com/article/10.3390/jpm13101502/s1, File S1: Protocols of the Reponses of the LLM and MTB.
