# Supplementary material for: Challenging ChatGPT 3.5 in Senology—An Assessment of Concordance with Breast Cancer Tumor Board Decision Making
Source: J Pers Med. 2023 Oct 16;13(10):1502. doi: 10.3390/jpm13101502 (PMC10608120; doi:10.3390/jpm13101502)
Supplement: Supplementary file 1 [file jpm-13-01502-s001.zip › File S1.pdf]

Randomization by https://www.random.org/sequences/
(performed on 30th of June 2023)

Randomized order of PP:

16
2
17
13
18
10
14
8
4
6
19
11
15
1
20
12
9
3
5
7

Supplementary S1.1: LLM´s Answers: p.2-p.61

Supplementary S1.2: MTB´s Answers to blinded German version: p.62-101

Supplementary S1.1: LLM´s Answers

P16

How should the following breast cancer patient be treated based on the most relevant and current international literature?

Patient information:

**Age:**	35
**Menopause status:**	premenopausal
**ECOG:**	0
**Previous illness:**	Paranoid schizophrenia (under stable condition with current olanzapine medication)
**Previous surgical treatment:**	none
**Birth history:**	no prior birth
**Oncological family history:**	Maternal grandmother with endometrial cancer at age of 60, paternal uncle with rectum carcinoma at the age of 50

The patient did not undergo surgery so far.

Following the surgical biopsy and staging, the following information about the tumor is available:

**TNM:**	cT2pN+pM1 (HEP)
**Histological classification:**	NST
**Grading:**	G3
**Estrogen receptor (ER):**	1%
**Progesterone receptor (PR):**	2%
**Her2-status:**	Negative (IHC 1+)
**Ki-67-proliferation-index:**	80%

Please provide a step-by-step treatment recommendation with regard to the necessity of neoadjuvant treatment, present or future surgical treatment, endocrine treatment, systemic treatment and radiation therapy taking the given patient information into consideration.

If endocrine treatment is advisable, please provide a suitable treatment regimen.

If systemic treatment is advisable, please provide a suitable treatment regimen.

If radiation therapy is advisable, please provide a suitable treatment regimen.

Based on the oncological family history, please state whether genetic testing should be performed or not.

ANSWER:

Based on the provided patient information and the most relevant and current international literature up to my knowledge cutoff date in September 2021, the treatment recommendation for the breast cancer patient can be as follows:

Step 1: Neoadjuvant Treatment Given the patient's age, premenopausal status, and the aggressive nature of the tumor (high-grade, high Ki-67 proliferation index), neoadjuvant treatment may be considered. Neoadjuvant treatment involves administering systemic therapy (chemotherapy or endocrine therapy) before surgery with the aim of reducing the tumor size and improving the chances of successful surgical resection.

Step 2: Genetic Testing Due to the patient's oncological family history (maternal grandmother with endometrial cancer and paternal uncle with rectal carcinoma), it is advisable to consider genetic testing for hereditary cancer syndromes. Genetic testing may identify potential germline mutations in cancer-associated genes like BRCA1, BRCA2, or others. Knowledge of these mutations could impact treatment decisions and may also have implications for the patient's family members.

Step 3: Endocrine Treatment Based on the tumor's hormone receptor status (ER 1% and PR 2%), the tumor is considered hormone receptor-positive (HR+). Endocrine therapy is recommended for HR+ breast cancer. However, the decision to use endocrine therapy may depend on the results of genetic testing (e.g., BRCA mutations) and the patient's preference for fertility preservation.

Suitable endocrine treatment regimen:

- Premenopausal patients: Tamoxifen is an option, which can be given for 5-10 years.
- In some cases, ovarian suppression or ablation may be added to tamoxifen to induce menopause and enhance the efficacy of endocrine therapy.

Step 4: Systemic Treatment As the HER2 status is negative (HER2-negative, IHC 1+), anti-HER2 targeted therapy is not indicated. However, due to the high grade and aggressive nature of the tumor, systemic chemotherapy is recommended as part of the neoadjuvant treatment.

Suitable systemic chemotherapy regimen:

- An appropriate chemotherapy regimen for a high-grade tumor may consist of anthracycline and taxane-based therapy (e.g., AC-T regimen: Doxorubicin and Cyclophosphamide followed by Paclitaxel).
- The specific chemotherapy regimen should be determined by the treating oncologist based on the patient's overall health and potential genetic factors.

Step 5: Radiation Therapy After neoadjuvant treatment and surgery, radiation therapy may be considered based on the pathological response to neoadjuvant treatment, the extent of tumor remaining after surgery, and other factors. The decision regarding radiation therapy should be made in consultation with a radiation oncologist.

It's essential to note that treatment decisions in cancer are complex and depend on various factors, including the patient's overall health, preferences, and specific tumor characteristics. Therefore, the patient's case should be discussed with a multidisciplinary team of oncologists to determine the most appropriate and personalized treatment plan.

Since medical knowledge and guidelines may have evolved beyond my last update in September 2021, I recommend consulting the latest international literature and guidelines for the most current treatment recommendations for breast cancer.

P2

How should the following breast cancer patient be treated based on the most relevant and current international literature?

Patient information:

**Age:**	61
**Menopause status:**	postmenopausal
**ECOG:**	0
**Previous illness:**	Hypothyroidism (with L-thyroxine medication)
**Previous surgical treatment:**	Open cholecystectomy at the age of 35, breast-conserving tumorectomy for right-sided fibroadenoma at the age of 32, uterine curettage after early abortion at age of 20
**Birth history:**	4 vaginal births at the age of 25, 27, 29 and 30, 1 early abortion at the age of 20
**Oncological family history:**	Maternal female cousin with hodgkin lymphoma at the age of 30

The patient underwent the following surgical treatment: Breast-conserving tumorectomy with sentinellymphnodectomy on the left side

Following the surgical treatment, the following TNM-classification, resection margin, histological classification, Grading and immunohistology were identified:

**TNM:**	pT2(2)pN1aM0
**Resection margin:**	R0, minimal resection margin of 6 mm
**Histological classification:**	Invasive-lobular
**Grading:**	G2
**Multifocality/-centricity:**	Monocentric and multifocal, 2 foci
**Estrogen receptor (ER):**	85%
**Progesterone receptor (PR):**	80%
**Her2-status:**	Negative (IHC 1+)
**Ki-67-proliferation-index:**	15%

Please provide a step-by-step treatment recommendation with regard to further surgical treatment, endocrine treatment, systemic treatment and radiation therapy taking the given patient information in consideration.

If endocrine treatment is advisable, please provide a suitable treatment regimen.

If systemic treatment is advisable, please provide a suitable treatment regimen.

If radiation therapy is advisable, please provide a suitable treatment regimen.

Based on the oncological family history, please state whether genetic testing should be performed or not.

ANSWER:

Based on the provided patient information and the most relevant and current international literature, the treatment recommendation for the breast cancer patient would be as follows:

1. **Endocrine Treatment:** Given the patient's postmenopausal status and the fact that the tumor is estrogen receptor (ER) and progesterone receptor (PR) positive, endocrine treatment is advisable. The standard endocrine treatment for this patient would be hormonal therapy with an aromatase inhibitor (AI). AIs are the preferred option in postmenopausal women with hormone receptor-positive breast cancer. The recommended regimen would be:
- Aromatase Inhibitor (AI): Letrozole or Anastrozole 1 mg orally daily for 5 years.
2. **Systemic Treatment:** The patient has invasive lobular breast cancer with positive lymph nodes (pT2(2)pN1aM0), indicating an intermediate risk for recurrence. Therefore, adjuvant systemic treatment with chemotherapy is recommended to reduce the risk of recurrence and improve overall survival. Based on the patient's health status and risk profile, the following chemotherapy regimen is suitable:
- Chemotherapy: The combination of an anthracycline (e.g., doxorubicin) and a taxane (e.g., paclitaxel) every 3 weeks for 4 to 6 cycles.
3. **Radiation Therapy:** The patient underwent breast-conserving tumorectomy, making her eligible for adjuvant radiation therapy. Radiation therapy is recommended to reduce the risk of local recurrence. The suitable treatment regimen is:
- Radiation Therapy: Whole breast radiation therapy with a standard fractionation scheme (e.g., 50 Gy in 25 fractions over 5 weeks).
4. **Genetic Testing:** Given the patient's family history of hodgkin lymphoma in a maternal female cousin at the age of 30, there may be a concern for hereditary cancer predisposition. In this case, it would be advisable to perform genetic testing to check for any genetic mutations that may increase the risk of breast cancer or other malignancies. The most common genes associated with hereditary breast cancer are BRCA1 and BRCA2. Genetic counseling and testing should be offered to the patient to assess her risk and make informed decisions about her future cancer management and surveillance.
5. **Follow-up and Monitoring:** After completion of the treatment regimens, the patient should be closely monitored with regular follow-up visits, mammograms, and other appropriate imaging and blood tests to detect any signs of recurrence or treatment-related side effects.

It is important to note that treatment decisions should always be made in consultation with the patient's oncologist, who can take into account individual

factors and preferences. The recommendations provided above are based on the information provided and general guidelines for breast cancer treatment.

P17

How should the following ductal carcinoma in situ patient be treated based on the most relevant and current international literature?

Patient information:

**Age:**	70
**Menopause status:**	postmenopausal
**ECOG:**	2
**Previous illness:**	Arterial hypertension (with AT II antagonist medication)
**Previous surgical treatment:**	Vaginal hysterectomy with bilateral adnexectomy for uterine prolapse at the age of 55, transcatheter aortic valve implantation due to aortic valve stenosis at the age of 69
**Birth history:**	2 vaginal births at the age of 16 and 20
**Oncological family history:**	1 sister with peritoneal cancer at the age of 60, maternal grandmother with ovarian cancer at the age of 65

The patient underwent the following surgical treatment: Right-sided mastectomy

Following the surgical treatment, the following TNM-classification, resection margin, histological classification, immunohistology were identified:

**TNM:**	pTis (size of the lesion 4.3 cm)
**Resection margin:**	R0, minimal resection margin of 10 mm
**Estrogen receptor (ER):**	95%
**Progesterone receptor (PR):**	90%

Please provide a step-by-step treatment recommendation with regard to further surgical treatment, endocrine treatment, systemic treatment and radiation therapy taking the given patient information in consideration.

If endocrine treatment is advisable, please provide a suitable treatment regimen.

If systemic treatment is advisable, please provide a suitable treatment regimen.

If radiation therapy is advisable, please provide a suitable treatment regimen.

Based on the oncological family history, please state whether genetic testing should be performed or not.

ANSWER:

As an AI language model, I can provide some general guidelines for the treatment of ductal carcinoma in situ (DCIS) based on the information provided. However, please keep in mind that I am not a medical professional, and the best treatment plan for an individual patient should be determined by an oncologist or a healthcare team who can consider the patient's unique medical history, current health status, and specific characteristics of the DCIS. Additionally, medical knowledge and guidelines may have evolved beyond my last update in September 2021, so consulting the most recent medical literature and guidelines is essential.

Step-by-step treatment recommendation for DCIS:

1. Surgical treatment: The patient already underwent a right-sided mastectomy, which is an appropriate surgical approach for DCIS. Given the TNM classification (pTis) and the 10 mm R0 resection margin, the initial surgery appears to be successful in removing the tumor with clear margins.
2. Endocrine treatment: The high expression of both estrogen receptor (ER) and progesterone receptor (PR) in the tumor (ER: 95%, PR: 90%) suggests that the patient may benefit from endocrine therapy. Endocrine therapy aims to block the action of hormones that can promote cancer growth. In postmenopausal women, aromatase inhibitors or selective estrogen receptor modulators (SERMs) can be used.

Potential endocrine treatment regimen:

- Aromatase inhibitor: Anastrozole, Letrozole, or Exemestane
- SERM: Tamoxifen

The choice between aromatase inhibitors and SERMs may depend on the patient's overall health status, comorbidities, and preferences. The duration of endocrine therapy may vary but is often recommended for at least 5 years.

3. Systemic treatment: In DCIS, systemic treatments like chemotherapy are typically not required since the disease is confined to the milk ducts and has not invaded surrounding tissues or lymph nodes. However, if there are any indications that invasive cancer might be present (e.g., based on pathology findings after surgery), the patient's oncologist may consider systemic treatments such as chemotherapy or targeted therapies based on the specific characteristics of the tumor.
4. Radiation therapy: Given the large size of the lesion (4.3 cm), post-mastectomy radiation therapy may be considered to reduce the risk of local recurrence. However, the decision to proceed with radiation therapy should be made based on the assessment of the patient's individual risk factors, overall health, and potential benefits versus risks of radiation therapy.

5. Genetic testing: The patient's oncological family history is notable for a sister with peritoneal cancer and a maternal grandmother with ovarian cancer and genetic testing may be advisable. While this information may raise some concern for hereditary cancer syndromes, such as BRCA mutations, the decision to perform genetic testing should be discussed with a genetic counselor or a healthcare professional specialized in cancer genetics. They can assess the family history in detail and determine whether genetic testing is appropriate. If genetic testing is recommended and a mutation is identified, it could have implications for the patient's treatment plan and inform potential risk for other cancers.

In conclusion, the treatment of DCIS involves a multidisciplinary approach, considering surgery, endocrine therapy, radiation therapy (if indicated), and potential genetic testing. It's crucial for the patient to work closely with their healthcare team to develop a personalized treatment plan based on their specific medical history and characteristics of the DCIS.

P13

How should the following breast cancer patient be treated based on the most relevant and current international literature?

Patient information:

**Age:**	56
**Menopause status:**	postmenopausal
**ECOG:**	1
**Previous illness:**	Insulin-dependent diabetes mellitus type 2, obesity with BMI of 43, obstructive sleep apnea syndrome, secondary arterial hypertension (with ACE inhibitor medication)
**Previous surgical treatment:**	Abdominoplasty at the age of 50, bilateral mammary reduction mammoplasty for mammary hypertrophy at the age of 35
**Birth history:**	3 vaginal births at the age of 20, 21 and 25
**Oncological family history:**	Mother with breast cancer at the age of 40

The patient underwent the following surgical treatment: Breast-conserving tumorectomy with sentinellymphnodectomy on the left side

Following the surgical treatment, the following TNM-classification, resection margin, histological classification, immunohistology were identified:

**TNM:**	pT1apN0M0
**Resection margin:**	R0, minimal resection margin of 1 mm
**Histological classification:**	NST
**Grading:**	G2
**Multifocality/-centricity:**	Monofocal and -centric
**Estrogen receptor (ER):**	0%
**Progesterone receptor (PR):**	0%
**Her2-status:**	Negative (IHC 1+)
**Ki-67-proliferation-index:**	40%

Please provide a step-by-step treatment recommendation with regard to further surgical treatment, endocrine treatment, systemic treatment and radiation therapy taking the given patient information in consideration.

If endocrine treatment is advisable, please provide a suitable treatment regimen.

If systemic treatment is advisable, please provide a suitable treatment regimen.

If radiation therapy is advisable, please provide a suitable treatment regimen.

Based on the oncological family history, please state whether genetic testing should be performed or not.

ANSWER:

Based on the provided patient information and the most relevant and current international literature, the treatment recommendation for the breast cancer patient is as follows:

1. Surgical Treatment: The patient has already undergone breast-conserving tumorectomy with sentinel lymph node dissection on the left side, and the resection margin was clear (R0). Since the tumor is monofocal and -centric, further surgical treatment is not immediately required.
2. Endocrine Treatment: The patient's tumor is estrogen receptor (ER) and progesterone receptor (PR) negative (both 0%). Therefore, endocrine therapy (hormone therapy) is not indicated in this case.
3. Systemic Treatment: Based on the provided information, the tumor is classified as NST (No Special Type) with a histological grade of G2. The Ki-67 proliferation index is high at 40%. The HER2 status is negative (IHC 1+).

   Considering the high Ki-67 proliferation index and the patient's postmenopausal status, systemic treatment with chemotherapy is advisable. Anthracycline/taxane-based chemotherapy regimens are commonly used in this setting. A common chemotherapy regimen for breast cancer patients with similar characteristics is "AC-T" (Adriamycin, Cyclophosphamide followed by Taxol).
4. Radiation Therapy: Radiation therapy is typically recommended after breast-conserving surgery to reduce the risk of local recurrence. Given that the patient underwent breast-conserving tumorectomy, she should receive adjuvant radiation therapy. The radiation therapy regimen will be determined by the radiation oncologist based on the extent of the original tumor and other factors.
5. Genetic Testing: Considering the patient's family history of breast cancer (mother with breast cancer at age 40), genetic testing for BRCA1 and BRCA2 mutations is advisable. Genetic testing may help identify if the patient has inherited a genetic mutation that increases her risk of developing breast and other related cancers. The decision for genetic testing should be discussed with a genetic counselor to assess the patient's risk and determine appropriate management if a mutation is identified.

In summary, the step-by-step treatment recommendation for the breast cancer patient is as follows:

1. No further surgical treatment is required at this time.
2. Endocrine treatment (hormone therapy) is not indicated due to ER and PR negative tumor.
3. Systemic treatment with AC-T chemotherapy is recommended based on the tumor characteristics.

4. Adjuvant radiation therapy should be administered after breast-conserving surgery.
5. Genetic testing for BRCA1 and BRCA2 mutations is advisable due to the family history of breast cancer.

Please note that individual patient cases may vary, and the treatment plan should be discussed and tailored by a multidisciplinary team of oncologists considering the patient's overall health status and specific characteristics.

P18

How should the following ductal carcinoma in situ patient be treated based on the most relevant and current international literature?

Patient information:

**Age:**	38
**Menopause status:**	premenopausal
**ECOG:**	0
**Previous illness:**	AV-node re-entry tachycardia (with beta-blocker medication)
**Previous surgical treatment:**	none
**Birth history:**	1 cesarean at the age of 36
**Oncological family history:**	Paternal grandfather with prostate cancer at the age of 65, mother with chronic myeloid leukemia at the age of 70

The patient underwent the following surgical treatment: Breast-conserving tumorectomy on the right and left side

Following the surgical treatment, the following TNM-classification, resection margin, histological classification and immunohistology were identified:

**TNM:**	pTis on left and right side (size of the lesions: 2.3 cm on left and 3.2 cm on right side)
**Resection margin:**	R0, minimal resection margin of 4 mm on left side and 5 mm on right side
**Estrogen receptor (ER):**	100% on left and right side
**Progesterone receptor (PR):**	100% on left and right side

Please provide a step-by-step treatment recommendation with regard to further surgical treatment, endocrine treatment, systemic treatment and radiation therapy taking the given patient information in consideration.

If endocrine treatment is advisable, please provide a suitable treatment regimen.

If systemic treatment is advisable, please provide a suitable treatment regimen.

If radiation therapy is advisable, please provide a suitable treatment regimen.

Based on the oncological family history, please state whether genetic testing should be performed or not.

ANSWER:

As an AI language model, I don't have access to the most up-to-date medical literature beyond my knowledge cutoff date in September 2021. Therefore, I cannot provide the absolute latest recommendations. However, I can offer a general guideline for the management of ductal carcinoma in situ (DCIS) based on the information provided up until my last update.

1. Surgical Treatment: The patient has already undergone breast-conserving tumorectomy on both sides (right and left). As both resection margins (R0) are clear with minimal distance, no further surgical treatment appears to be needed.
2. Endocrine Treatment: Given that the patient is premenopausal and the tumor is ER-positive (100% on both sides), endocrine therapy may be recommended. The most common endocrine treatment in this case would be tamoxifen. Tamoxifen is an anti-estrogen medication that can reduce the risk of DCIS recurrence and contralateral breast cancer. The treatment duration typically lasts for five years, but the specific duration should be discussed with the patient's oncologist based on individual factors.
3. Systemic Treatment: Systemic treatment, such as chemotherapy, is not typically indicated for pure DCIS cases, as it is considered a non-invasive stage of breast cancer. However, if there is an invasive component or other high-risk features, systemic treatment may be considered. Since the provided information only mentions ductal carcinoma in situ with no mention of invasive carcinoma, systemic treatment might not be necessary at this stage.
4. Radiation Therapy: Radiation therapy is usually recommended after breast-conserving surgery to reduce the risk of local recurrence. Considering the tumor size and involvement on both sides (2.3 cm on the left and 3.2 cm on the right), radiation therapy is likely to be recommended for this patient. The radiation regimen will be determined by the radiation oncologist based on individual factors.
5. Genetic Testing: Given the patient's family history of cancer (prostate cancer in the paternal grandfather and chronic myeloid leukemia in the mother), genetic testing may be considered. BRCA1 and BRCA2 gene mutations, in particular, are associated with an increased risk of breast cancer and may warrant genetic testing. A genetic counselor can help assess the appropriateness and implications of genetic testing for this patient.

It is important to remember that each patient's case is unique, and treatment decisions should be made in consultation with a multidisciplinary team of healthcare professionals, including oncologists, surgeons, radiation oncologists, and genetic counselors, based on the most current and relevant information available at the time of treatment. For the latest and most accurate recommendations, it is essential to

consult with a medical professional who has access to up-to-date literature and patient-specific details.

P10

How should the following breast cancer patient be treated based on the most relevant and current international literature?

Patient information:

**Age:**	65
**Menopause status:**	postmenopausal
**ECOG:**	2
**Previous illness:**	Atrial fibrillation (with direct oral anticoagulant and beta-blocker medication), pulmonary artery embolism at the age of 65 following immobilization during right-sided total hip arthroplasty
**Previous surgical treatment:**	Right-sided total hip arthroplasty at the age of 65
**Birth history:**	2 vaginal births at the age of 23 and 30 and 1 cesarean at the age of 35
**Oncological family history:**	Sister with childhood acute lymphoblastic leukemia, father with gastric carcinoma at the age of 50

The patient underwent the following surgical treatment: Mastectomy with sentinellymphnodectomy on the right side

Following the surgical treatment, the following TNM-classification, resection margin, histological classification, Grading and immunohistology were identified:

**TNM:**	pT3pN1aM0
**Resection margin:**	R0, minimal resection margin of 10 mm
**Histological classification:**	NST
**Grading:**	G2
**Multifocality/-centricity:**	Monofocal and -centric
**Estrogen receptor (ER):**	0%
**Progesterone receptor (PR):**	0%
**Her2-status:**	Positive (ICH 3+)
**Ki-67-proliferation-index:**	35%

Please provide a step-by-step treatment recommendation with regard to further surgical treatment, endocrine treatment, systemic treatment and radiation therapy taking the given patient information in consideration.

If endocrine treatment is advisable, please provide a suitable treatment regimen.

If systemic treatment is advisable, please provide a suitable treatment regimen.

If radiation therapy is advisable, please provide a suitable treatment regimen.

Based on the oncological family history, please state whether genetic testing should be performed or not.

ANSWER:

As an AI language model, I can provide general guidelines based on the information you provided, but it's important to remember that treatment decisions for breast cancer are complex and should be made by a multidisciplinary team of healthcare professionals, taking into account the patient's individual characteristics and preferences. Additionally, my knowledge is based on information available up to September 2021, and there may be more recent developments in breast cancer treatment. With these caveats in mind, here is a step-by-step treatment recommendation for the patient:

Step 1: Further Surgical Treatment The patient already underwent mastectomy with sentinel lymph node dissection, which is a standard surgical treatment for breast cancer. Since the surgical resection margins were clear (R0) with a minimal margin of 10 mm, further surgical intervention is not immediately required.

Step 2: Systemic Treatment Based on the histological classification and molecular characteristics of the tumor, systemic treatment is advisable for this patient.

a) Endocrine Treatment: The patient's tumor is ER and PR negative, which means it is hormone receptor-negative. Endocrine therapy is not effective in hormone receptor-negative breast cancer. Therefore, endocrine treatment is not applicable in this case.

b) HER2-Targeted Therapy: The patient's tumor is HER2-positive (HER2 3+), indicating a higher expression of the HER2 protein. HER2-targeted therapy is recommended in addition to chemotherapy.

c) Chemotherapy: Given the TNM classification (pT3pN1aM0), which suggests that the tumor is larger (T3) with regional lymph node involvement (N1a) but no distant metastasis (M0), adjuvant chemotherapy is appropriate to reduce the risk of recurrence and improve overall survival.

Step 3: Systemic Treatment Regimen The most suitable systemic treatment regimen for this patient would likely include a combination of HER2-targeted therapy and chemotherapy. An appropriate regimen could be:

- HER2-Targeted Therapy: Trastuzumab, a monoclonal antibody targeting HER2, is the standard of care for HER2-positive breast cancer. It is usually given every three weeks for a total of one year (approximately 18 cycles).
- Chemotherapy: A common chemotherapy regimen for HER2-positive breast cancer is a combination of docetaxel, carboplatin (or cisplatin), and trastuzumab. The chemotherapy is usually given in cycles every three weeks for a total of four to six cycles.

The specific chemotherapy drugs and dosages should be determined by the oncologist based on the patient's overall health and tolerance to chemotherapy.

Step 4: Radiation Therapy Given the patient's TNM classification (pT3pN1aM0) and the fact that she underwent mastectomy with sentinel lymph node dissection, radiation therapy may be considered to further reduce the risk of local recurrence. Radiation therapy is typically given after surgery when there is a higher risk of local recurrence.

Step 5: Genetic Testing Considering the patient's oncological family history, which includes a sister with childhood acute lymphoblastic leukemia and a father with gastric carcinoma, it may be appropriate to perform genetic testing for hereditary cancer syndromes. Genetic counseling and testing for BRCA1, BRCA2, and other relevant genes associated with breast cancer may be considered to assess the patient's risk of developing other malignancies and to inform treatment decisions.

In summary, the recommended treatment plan for this postmenopausal breast cancer patient with the provided information would likely involve HER2-targeted therapy, chemotherapy, and possibly radiation therapy. Genetic testing for hereditary cancer syndromes should also be considered to assess the patient's risk profile. It's important for the patient to discuss these recommendations with an oncologist who can tailor the treatment plan based on her individual circumstances and preferences.

P14

How should the following breast cancer patient be treated based on the most relevant and current international literature?

Patient information:

**Age:**	65
**Menopause status:**	postmenopausal
**ECOG:**	1
**Previous illness:**	Addison's disease (currently under hydro- and fludrocortisone medication)
**Previous surgical treatment:**	Total knee replacement on the left side at the age of 50
**Birth history:**	3 cesareans at the age of 25, 28 and 35
**Oncological family history:**	Paternal grandmother with pancreatic cancer at the age of 59, maternal aunt with colon cancer at the age of 60

The patient did not undergo surgery so far.

Following the surgical biopsy and staging, the following information about the tumor is available:

**TNM:**	cT3pN+pM1 (OSS)
**Histological classification:**	NST
**Grading:**	G3
**Estrogen receptor (ER):**	0%
**Progesterone receptor (PR):**	0%
**Her2-status:**	Negative (IHC 0)
**Ki-67-proliferation-index:**	60%

Please provide a step-by-step treatment recommendation with regard to the necessity of neoadjuvant treatment, present or future surgical treatment, endocrine treatment, systemic treatment and radiation therapy taking the given patient information into consideration.

If endocrine treatment is advisable, please provide a suitable treatment regimen.

If systemic treatment is advisable, please provide a suitable treatment regimen.

If radiation therapy is advisable, please provide a suitable treatment regimen.

Based on the oncological family history, please state whether genetic testing should be performed or not.

ANSWER:

Based on the provided patient information and the characteristics of the tumor, here is a step-by-step treatment recommendation for this breast cancer patient:

Step 1: Neoadjuvant Treatment Given the locally advanced nature of the tumor (cT3) and positive lymph nodes (cN+), neoadjuvant treatment is advisable. Neoadjuvant treatment involves administering systemic therapy (chemotherapy and/or endocrine therapy) before surgery to downsize the tumor and potentially increase the likelihood of successful surgical resection.

Step 2: Endocrine Treatment The tumor is negative for both estrogen receptors (ER) and progesterone receptors (PR), which means it is hormone receptor-negative. Since the patient is postmenopausal, endocrine therapy may not be effective in this case, as the tumor does not rely on estrogen or progesterone for growth. Therefore, endocrine therapy is not advisable in this scenario.

Step 3: Systemic Treatment The tumor is classified as NST (invasive carcinoma of no special type) and is grade 3, indicating aggressive behavior. The Ki-67 proliferation index is high at 60%, which further indicates a high rate of cell division and aggressiveness. The HER2 status is negative, ruling out targeted therapy with anti-HER2 agents.

In this case, systemic treatment with chemotherapy is the mainstay of neoadjuvant therapy. A suitable treatment regimen could be a combination of anthracycline and taxane-based chemotherapy, such as FEC-T (5-fluorouracil, epirubicin, cyclophosphamide, followed by docetaxel) or AC-T (doxorubicin, cyclophosphamide, followed by paclitaxel). The specific chemotherapy regimen and dosages should be determined by the patient's oncologist based on their overall health and medical history.

Step 4: Surgical Treatment Following neoadjuvant chemotherapy, the tumor's response to treatment should be evaluated. Depending on the tumor's size and extent of response, the patient may be a candidate for breast-conserving surgery (lumpectomy) or mastectomy. The decision between these surgical options will be made by the surgeon based on the patient's preference, tumor size, and location, among other factors.

Step 5: Radiation Therapy After surgery, radiation therapy may be recommended to further reduce the risk of local recurrence. The decision for radiation therapy will depend on the extent of the initial tumor and whether breast-conserving surgery or mastectomy was performed. The radiation therapy plan will be determined by a radiation oncologist.

Genetic Testing: Given the patient's oncological family history with pancreatic cancer in the paternal grandmother and colon cancer in the maternal aunt, there might be a

potential hereditary component to the breast cancer. As such, genetic testing for BRCA1 and BRCA2 mutations should be considered in this patient. BRCA gene mutations can increase the risk of breast and ovarian cancer. Genetic counseling and testing should be offered to the patient to assess hereditary risk and guide further management and screening recommendations for both the patient and her family members.

Please note that treatment decisions in cancer care are complex and require a multidisciplinary approach. The patient's oncologist and healthcare team will make the final treatment recommendations based on the most relevant and current international literature and the patient's individual circumstances

P8

How should the following breast cancer patient be treated based on the most relevant and current international literature?

Patient information:

**Age:**	35
**Menopause status:**	premenopausal
**ECOG:**	0
**Previous illness:**	Colitis ulcerosa, Hashimoto-thyreoiditis (with L-thyroxine medication)
**Previous surgical treatment:**	Laparoscopy for cyst extirpation of left ovarian cyst at age 30
**Birth history:**	no prior birth
**Oncological family history:**	Paternal grandmother with breast cancer at the age of 70, paternal aunt with breast cancer at the age of 50, maternal uncle with pancreatic cancer at the age of 60

The patient underwent the following surgical treatment: Breast-conserving tumorectomy with sentinellymphnodectomy on the right side

Following the surgical treatment, the following TNM-classification, resection margin, histological classification, Grading and immunohistology were identified:

**TNM:**	pT2pN1cM0
**Resection margin:**	R0, minimal resection margin of 2 mm
**Histological classification:**	Invasive-lobular
**Grading:**	G3
**Multifocality/-centricity:**	Monofocal and -centric
**Estrogen receptor (ER):**	75%
**Progesterone receptor (PR):**	75%
**Her2-status:**	Positive (IHC 3+)
**Ki-67-proliferation-index:**	40%

Please provide a step-by-step treatment recommendation with regard to further surgical treatment, endocrine treatment, systemic treatment and radiation therapy taking the given patient information in consideration.

If endocrine treatment is advisable, please provide a suitable treatment regimen.

If systemic treatment is advisable, please provide a suitable treatment regimen.

If radiation therapy is advisable, please provide a suitable treatment regimen.

Based on the oncological family history, please state whether genetic testing should be performed or not.

ANSWER:

As an AI language model, I am not a medical professional and cannot provide personalized medical advice. Treatment decisions for breast cancer should always be made by a multidisciplinary team of healthcare professionals based on the patient's individual characteristics, medical history, and current condition, in conjunction with the most up-to-date international guidelines and literature. However, I can outline a general approach to the treatment of this breast cancer patient based on the information provided.

Step-by-step treatment recommendation:

1. Surgical Treatment: The patient has already undergone breast-conserving tumorectomy with sentinel lymph node dissection on the right side, and the resection margin was R0 (no tumor cells at the margin). This suggests that the primary tumor has been removed, and there is no immediate need for additional surgical treatment.
2. Endocrine Treatment: Given the positive hormone receptor status (ER 75% and PR 75%), endocrine therapy is recommended for this patient. As the patient is premenopausal, the most appropriate endocrine treatment would likely be tamoxifen, a selective estrogen receptor modulator (SERM). Tamoxifen is generally the standard of care for premenopausal women with hormone receptor-positive breast cancer. However, the specific duration and dosing of tamoxifen should be discussed with the patient's oncologist.
3. Systemic Treatment: The patient's breast cancer is Her2-positive (Her2 3+ by IHC), and it is considered invasive lobular (histological classification G3). With the high Ki-67 proliferation index (40%), the cancer appears to be aggressive. Therefore, systemic treatment, such as chemotherapy or targeted therapy, is likely to be recommended to address the aggressive nature of the tumor.

For Her2-positive breast cancer, targeted therapy with anti-Her2 agents is a standard treatment. Trastuzumab (Herceptin) is a common option and is typically given in combination with chemotherapy. The choice of chemotherapy regimen would depend on various factors, including the patient's overall health and preferences, but a common regimen is TCH (docetaxel, carboplatin, and trastuzumab).

4. Radiation Therapy: Given the tumor characteristics (pT2, pN1c), radiation therapy may be recommended to reduce the risk of local recurrence. The precise radiation treatment plan (dose, duration, etc.) would be determined by the radiation oncologist based on the specific clinical factors.
5. Genetic Testing: Given the oncological family history with multiple relatives affected by cancer, genetic testing for hereditary breast and ovarian cancer syndrome may be considered. BRCA1 and BRCA2 gene mutations are associated with an increased risk of breast cancer. Testing for other relevant genetic mutations may also be warranted. The decision for genetic testing

should be made in consultation with a genetic counselor or a healthcare professional with expertise in cancer genetics.

It is important to stress that the above recommendations are general and should not be taken as definitive treatment advice for this particular patient. Treatment plans may vary based on the patient's overall health, individual circumstances, and the latest clinical guidelines and research at the time of the patient's evaluation. The patient should discuss their specific case with their oncologist to formulate the most appropriate and personalized treatment plan.

P4

How should the following breast cancer patient be treated based on the most relevant and current international literature?

Patient information:

**Age:**	45
**Menopause status:**	premenopausal
**ECOG:**	1
**Previous illness:**	HELLP Syndrome at first pregnancy at age of 34
**Previous surgical treatment:**	Postpartum cardiomyopathy with intensive care ECMO support, Roux-Y gastric bypass for obesity (BMI 50) at the age of 32
**Birth history:**	2 cesareans at the age of 34 and 38
**Oncological family history:**	Paternal uncle with prostate cancer at the age of 65

The patient underwent the following surgical treatment: Breast-conserving tumorectomy with sentinellymphnodectomy on the left side

Following the surgical treatment, the following TNM-classification, resection margin, histological classification, Grading and immunohistology were identified:

**TNM:**	pT1cpN1aM0
**Resection margin:**	R1 on lateral aspect
**Histological classification:**	NST
**Grading:**	G2
**Multifocality/-centricity:**	Monofocal and -centric
**Estrogen receptor (ER):**	100%
**Progesterone receptor (PR):**	100%
**Her2-status:**	Negative (IHC 0)
**Ki-67-proliferation-index:**	10%

Please provide a step-by-step treatment recommendation with regard to further surgical treatment, endocrine treatment, systemic treatment and radiation therapy taking the given patient information in consideration.

If endocrine treatment is advisable, please provide a suitable treatment regimen.

If systemic treatment is advisable, please provide a suitable treatment regimen.

If radiation therapy is advisable, please provide a suitable treatment regimen.

Based on the oncological family history, please state whether genetic testing should be performed or not.

ANSWER:

As an AI language model, I can provide general guidelines based on the information given. However, it's crucial to remember that every breast cancer case is unique, and treatment decisions should be made by a multidisciplinary team of healthcare professionals who have access to the patient's complete medical history and can consider all relevant factors. These recommendations are based on the provided information and may not be exhaustive or up-to-date. Always consult with a qualified healthcare professional for personalized advice.

Step-by-step treatment recommendation for the breast cancer patient:

1. Surgical Treatment: The patient already underwent breast-conserving tumorectomy with sentinellymphnodectomy on the left side. However, it's important to address the R1 resection margin on the lateral aspect. A re-excision surgery or mastectomy may be considered to achieve clear margins. Consultation with a surgical oncologist is necessary to decide the best course of action.
2. Endocrine Treatment: Given the patient's ER and PR-positive status, endocrine therapy is recommended. As the patient is premenopausal, the standard treatment would be to offer ovarian function suppression in combination with an aromatase inhibitor. Alternatively, tamoxifen can be considered in combination with ovarian function suppression. The duration of endocrine therapy will depend on the patient's response and individual risk factors.
3. Systemic Treatment: The patient's tumor is HER2-negative and has a low Ki-67 proliferation index, indicating a lower risk of aggressive behavior. Therefore, systemic chemotherapy may not be indicated in this case. However, the decision to administer chemotherapy should be based on additional factors, such as the number of positive lymph nodes and the patient's overall health. A medical oncologist should assess the necessity of chemotherapy and discuss potential benefits and risks with the patient.
4. Radiation Therapy: Given the positive lymph nodes (N1a) and the positive resection margin (R1), adjuvant radiation therapy is recommended to reduce the risk of local recurrence. External beam radiation to the breast and regional lymph nodes is typically the standard approach. The dose and duration of radiation will be determined by a radiation oncologist.
5. Genetic Testing: The patient's oncological family history includes a paternal uncle with prostate cancer at the age of 65. There is not a strong indication for genetic testing. A genetic counselor can help assess the need for genetic testing based on the patient's family history and may recommend testing for specific breast cancer susceptibility genes like BRCA1 and BRCA2.

Please remember that these recommendations are general guidelines and may be subject to change based on the latest research and medical evidence. The patient

should work closely with their healthcare team to create a personalized treatment plan that considers their specific situation, preferences, and overall health.

P6

How should the following breast cancer patient be treated based on the most relevant and current international literature?

Patient information:

**Age:**	58
**Menopause status:**	postmenopausal
**ECOG:**	1
**Previous illness:**	Crohn's disease (with continuous therapy with TNF-alpha inhibitors)
**Previous surgical treatment:**	Bowel-sparing resection for ileum stenosis at the age of 35, open appendectomy at the age of 25, longitudinal laparotomy for mechanical ileus at the age of 55
**Birth history:**	no prior birth
**Oncological family history:**	Maternal grandmother with breast cancer at the age of 80

The patient underwent the following surgical treatment: Breast-conserving tumorectomy with sentinellymphnodectomy on the left side

Following the surgical treatment, the following TNM-classification, resection margin, histological classification, Grading and immunohistology were identified:

**TNM:**	pT3(3)pN1aM0
**Resection margin**:	R0, minimal resection margin of 7 mm
**Histological classification:**	NST
**Grading:**	G2
**Multifocality/-centricity:**	Monocentric and multifocal, 3 foci
**Estrogen receptor (ER):**	75%
**Progesterone receptor (PR):**	90%
**Her2-status:**	Negative (IHC 0)
**Ki-67-proliferation-index:**	28%

Please provide a step-by-step treatment recommendation with regard to further surgical treatment, endocrine treatment, systemic treatment and radiation therapy taking the given patient information in consideration.

If endocrine treatment is advisable, please provide a suitable treatment regimen.

If systemic treatment is advisable, please provide a suitable treatment regimen.

If radiation therapy is advisable, please provide a suitable treatment regimen.

Based on the oncological family history, please state whether genetic testing should be performed or not.

ANSWER:

As an AI language model, I am not a medical professional, and I cannot provide personalized treatment recommendations. However, I can offer general guidance based on the information provided and standard breast cancer treatment approaches as of my last update in September 2021. It's essential to consult with an oncologist who has access to the most up-to-date medical literature and knowledge of the patient's specific medical history before making any treatment decisions. Treatment plans may vary depending on the patient's overall health, preferences, and other individual factors.

Step-by-step treatment recommendation:

1. Surgical Treatment: The patient has already undergone breast-conserving tumorectomy with sentinel lymph node biopsy on the left side. Since the histological classification is NST (No Special Type) and the resection margin is 0 with a minimal resection margin of 7 mm, the surgical treatment appears to be complete.
2. Endocrine Treatment: Given the patient's hormone receptor status, which includes ER-positive (5%) and PR-positive (90%) tumor, endocrine therapy is recommended. Endocrine therapy is an effective treatment for hormone receptor-positive breast cancer.

   Suitable Treatment Regimen: The most appropriate endocrine therapy in postmenopausal women with hormone receptor-positive breast cancer is usually aromatase inhibitors (AI) or selective estrogen receptor modulators (SERMs). Anastrozole, letrozole, or exemestane are commonly used AIs.
3. Systemic Treatment: Considering the patient's tumor characteristics (pT3(3)pN1aM0, G2, Ki-67 proliferation index 28%, and Her2-negative), chemotherapy may be considered to reduce the risk of recurrence, especially with the presence of positive lymph nodes. The decision for systemic chemotherapy will depend on various factors, including the patient's overall health, preferences, and risk of recurrence.

   Suitable Treatment Regimen: The specific chemotherapy regimen, if recommended, will be determined by the oncologist based on the patient's health status, medical history, and other factors. Commonly used chemotherapy drugs for breast cancer include anthracyclines (e.g., doxorubicin or epirubicin) and taxanes (e.g., paclitaxel or docetaxel).
4. Radiation Therapy: Radiation therapy is often recommended after breast-conserving surgery to reduce the risk of local recurrence. Given the patient's T-stage (pT3), multifocality, and nodal involvement (pN1a), radiation therapy is likely to be advised.

   Suitable Treatment Regimen: The radiation therapy regimen will be determined by the radiation oncologist based on the patient's specific clinical

> characteristics. The treatment usually involves daily radiation sessions over a few weeks.

Genetic Testing: Based on the patient's oncological family history, which includes breast cancer in the maternal grandmother at the age of 80, genetic testing for hereditary breast cancer syndromes may be considered. A genetic counselor can help assess the patient's family history and provide appropriate recommendations for genetic testing. If the patient is found to have a pathogenic mutation, it may impact treatment decisions and potentially influence the treatment plan.

Please note that these treatment recommendations are based on the information provided and may not cover all potential aspects of the patient's case. The actual treatment plan must be determined by an oncologist after a thorough evaluation of the patient's specific medical condition and individual needs.

P19

How should the following ductal carcinoma in situ patient be treated based on the most relevant and current international literature?

Patient information:

**Age:**	72
**Menopause status:**	postmenopausal
**ECOG:**	1
**Previous illness:**	Chronic lymphocytic leukemia Stadium A
**Previous surgical treatment:**	Total shoulder arthroplasty on the left side
**Birth history:**	1 vaginal birth at the age of 22
**Oncological family history:**	Father with colon cancer at age 55

The patient underwent the following surgical treatment: Breast-conserving tumorectomy on the left side

Following the surgical treatment, the following TNM-classification, resection margin, histological classification and immunohistology were identified:

**TNM:**	pTis (size of lesion 1.5 cm)
**Resection margin:**	R0, with minimal resection margin of 0.01 mm
**Estrogen receptor (ER):**	100%
**Progesterone receptor (PR):**	100%

Please provide a step-by-step treatment recommendation with regard to further surgical treatment, endocrine treatment, systemic treatment and radiation therapy taking the given patient information in consideration.

If endocrine treatment is advisable, please provide a suitable treatment regimen.

If systemic treatment is advisable, please provide a suitable treatment regimen.

If radiation therapy is advisable, please provide a suitable treatment regimen.

Based on the oncological family history, please state whether genetic testing should be performed or not.

ANSWER:

As an AI language model, I don't have access to real-time data or the ability to browse the internet for the most current medical literature. My knowledge is based on information available up to September 2021. Therefore, I can provide general guidelines for the treatment of ductal carcinoma in situ (DCIS) based on the information you provided up to that date. However, for the most relevant and current treatment recommendations, it is essential to consult with an oncologist who has access to the latest research and guidelines.

Given the patient's information:

1. Age: 72
2. Menopause status: Postmenopausal
3. ECOG: 1 (Eastern Cooperative Oncology Group performance status of 1, indicating the patient is ambulatory and capable of light physical activity)
4. Previous illness: Chronic lymphocytic leukemia (Stadium A)
5. Previous surgical treatment: Total shoulder arthroplasty on the left side
6. Birth history: 1 vaginal birth at the age of 22
7. Oncological family history: Father with colon cancer at age 55

Surgical Treatment: The patient has already undergone breast-conserving tumorectomy on the left side, resulting in complete resection with clear margins (R0). This means that all detectable cancer cells were removed with a minimal resection margin of 0.01 mm. Since DCIS was diagnosed, it is crucial to ensure that all cancerous cells have been removed entirely.

Endocrine Treatment: Given that the hormone receptors (ER and PR) are both 100% positive, endocrine treatment with hormonal therapy might be considered as adjuvant therapy. The most common endocrine treatment for hormone receptor-positive DCIS in postmenopausal women is the use of selective estrogen receptor modulators (SERMs) like tamoxifen or aromatase inhibitors. However, the decision to proceed with endocrine treatment should be carefully discussed with the patient's oncologist, considering her overall health and the potential benefits and risks of the therapy.

Systemic Treatment: In general, systemic treatment (chemotherapy) is not indicated for pure DCIS, as it is a non-invasive cancer confined to the milk ducts. Systemic treatment is typically reserved for invasive breast cancer cases.

Radiation Therapy: Radiation therapy is commonly recommended following breast-conserving surgery for DCIS to reduce the risk of local recurrence. However, the decision to proceed with radiation therapy depends on various factors, including the size of the tumor, the extent of surgical resection, and the patient's overall health. Since the tumor size is 1.5 cm (pTis), radiation therapy might be considered.

Genetic Testing: The patient's family history of colon cancer in her father at age 55 is not directly related to breast cancer. However, considering that there might be some familial cancer predisposition, discussing genetic testing with a genetic counselor could be beneficial to assess the patient's risk of having an underlying genetic mutation, such as BRCA1 or BRCA2, which are associated with an increased risk of breast cancer.

It's important to reiterate that treatment decisions should be made in consultation with the patient's oncologist, who will consider the patient's individual characteristics, the most current guidelines, and the latest available literature to determine the best course of action. Cancer treatment is highly individualized, and a multidisciplinary team approach is often employed to ensure the best outcome for the patient.

P11

How should the following breast cancer patient be treated based on the most relevant and current international literature?

Patient information:

**Age:**	32
**Menopause status:**	premenopausal
**ECOG:**	0
**Previous illness:**	Insulin-dependent gestational diabetes during the first pregnancy at the age of 20, postpartum depression at the age of 20
**Previous surgical treatment:**	none
**Birth history:**	1 vaginal birth at the age of 20
**Oncological family history:**	Father with bronchial carcinoma at the age of 60, sister with osteosarcoma at the age of 18

The patient underwent the following surgical treatment: Breast-conserving tumorectomy with sentinellymphnodectomy on the left side

Following the surgical treatment, the following TNM-classification, resection margin, histological classification, Grading and immunohistology were identified:

**TNM:**	pT2pN0M0
**Resection margin:**	R0, minimal resection margin of 4 mm
**Histological classification:**	NST
**Grading:**	G2
**Multifocality/-centricity:**	Monofocal and -centric
**Estrogen receptor (ER):**	0%
**Progesterone receptor (PR):**	0%
**Her2-status:**	Positive (ICH 3+)
**Ki-67-proliferation-index:**	65%

Please provide a step-by-step treatment recommendation with regard to further surgical treatment, endocrine treatment, systemic treatment and radiation therapy taking the given patient information in consideration.

If endocrine treatment is advisable, please provide a suitable treatment regimen.

If systemic treatment is advisable, please provide a suitable treatment regimen.

If radiation therapy is advisable, please provide a suitable treatment regimen.

Based on the oncological family history, please state whether genetic testing should be performed or not.

ANSWER:

As an AI language model, I am not a medical professional, and my responses should not be considered as a substitute for professional medical advice. Treatment decisions for breast cancer patients should always be made by qualified healthcare professionals based on the patient's individual circumstances, medical history, and current medical literature.

That being said, I can provide some general information about the possible treatment options for the breast cancer patient described.

Step-by-step treatment recommendation:

1. Further Surgical Treatment: The patient has already undergone breast-conserving tumorectomy with sentinel lymph node dissection on the left side, and the resection margin is reported as R0 (indicating clear margins). At this stage, there is no evidence to suggest additional surgical treatment. The next steps in the treatment plan will typically involve adjuvant therapies to reduce the risk of cancer recurrence.
2. Endocrine Treatment: Given that the patient has a hormone receptor-negative breast cancer (ER 0% and PR 0%), endocrine therapy may not be applicable in this case.
3. Systemic Treatment: The patient's breast cancer is HER2-positive (HER2 3+ by immunohistochemistry), and the Ki-67 proliferation index is high (65%). Considering the high Ki-67 proliferation index and HER2-positive status, systemic treatment with chemotherapy and targeted therapy (anti-HER2 therapy) is usually recommended to reduce the risk of recurrence and improve survival.

Suitable Treatment Regimen: The most current and relevant international literature should be consulted to determine the most appropriate chemotherapy and anti-HER2 therapy regimen for the patient. Commonly used chemotherapy drugs for HER2-positive breast cancer include a combination of anthracycline and taxane-based regimens. The anti-HER2 targeted therapy can include drugs like trastuzumab, pertuzumab, or ado-trastuzumab emtansine (T-DM1). The choice of regimen will depend on the patient's overall health, potential side effects, and specific guidelines from the patient's country or region.

4. Radiation Therapy: After breast-conserving tumorectomy, adjuvant radiation therapy is typically recommended to reduce the risk of local recurrence. The radiation therapy plan will depend on the extent of the initial tumor and other factors that the radiation oncologist will consider.
5. Genetic Testing: Based on the oncological family history of breast cancer in the patient's father and sister, there may be a concern for potential hereditary breast cancer predisposition. Therefore, genetic testing and counseling for

BRCA1 and BRCA2 mutations and other relevant genetic mutations may be advisable to assess the patient's risk and help inform further treatment decisions. A genetic counselor or oncologist experienced in cancer genetics can guide the patient through the testing process and its implications.

It's crucial for the patient to work closely with an oncology team to tailor the treatment plan to their specific situation. The treatment recommendations should be based on the most recent international guidelines and research, and the patient's preferences and overall health should be taken into account.

P15

How should the following breast cancer patient be treated based on the most relevant and current international literature?

Patient information:

**Age:**	29
**Menopause status**:	premenopausal
**ECOG:**	0
**Previous illness:**	none
**Previous surgical treatment:**	none
**Birth history:**	no prior birth
**Oncological family history:**	Mother with breast cancer at the age of 65, maternal grandmother with breast cancer at the age of 70

The patient did not undergo surgery so far.

Following the surgical biopsy and staging, the following information about the tumor is available:

**TNM:**	cT2cN0M0 on left side and cT1bcN0M0 on right side
**Histological classification:**	NST on left and right side
**Grading:**	G3
**Estrogen receptor (ER):**	0% on left and right side
**Progesterone receptor (PR):**	0% on left and right side
**Her2-status:**	Negative (IHC 0) on left and right side
**Ki-67-proliferation-index:**	70% on left side, 85% on right side

Please provide a step-by-step treatment recommendation with regard to the necessity of neoadjuvant treatment, present or future surgical treatment, endocrine treatment, systemic treatment and radiation therapy taking the given patient information into consideration.

If endocrine treatment is advisable, please provide a suitable treatment regimen.

If systemic treatment is advisable, please provide a suitable treatment regimen.

If radiation therapy is advisable, please provide a suitable treatment regimen.

Based on the oncological family history, please state whether genetic testing should be performed or not.

ANSWER:

Based on the patient's information, here is a step-by-step treatment recommendation for breast cancer:

Step 1: Neoadjuvant Treatment (Preoperative Systemic Therapy) Given the patient's young age, aggressive tumor features (high-grade, high Ki-67 proliferation index), and the large size of the tumor (cT2cN0M0 on the left side), neoadjuvant treatment may be advisable to reduce the tumor size and increase the chances of successful surgical outcomes.

Regimen for Neoadjuvant Systemic Treatment: The most appropriate neoadjuvant systemic treatment regimen for this patient would be a combination of chemotherapy. Given the tumor's triple-negative (ER-, PR-, and HER2-) status, chemotherapy is the standard treatment option.

Recommended Chemotherapy Regimen: A commonly used and effective chemotherapy regimen for triple-negative breast cancer is a combination of anthracycline and taxane-based chemotherapy. For example, a regimen with dose-dense doxorubicin and cyclophosphamide followed by paclitaxel would be a suitable choice.

Step 2: Surgical Treatment After completion of neoadjuvant chemotherapy, the patient should be reassessed for surgical intervention. Depending on the response to neoadjuvant treatment, the surgical options can vary.

Left-Side Tumor: Since the left-side tumor is larger (cT2c), breast-conserving surgery (lumpectomy) may not be suitable. A mastectomy (total removal of the breast) would be the preferred surgical approach.

Right-Side Tumor: As the right-side tumor is smaller (cT1b), a breast-conserving surgery (lumpectomy) might be considered if the tumor has responded well to neoadjuvant chemotherapy.

Step 3: Adjuvant Systemic Treatment Given the high-grade nature and triple-negative status of the tumors, adjuvant chemotherapy should be considered to reduce the risk of recurrence after surgery.

Recommended Adjuvant Chemotherapy Regimen: A standard adjuvant chemotherapy regimen for triple-negative breast cancer includes a combination of anthracycline and taxane-based chemotherapy, similar to the neoadjuvant regimen. The choice of specific drugs may be adjusted based on the patient's response to neoadjuvant treatment.

Step 4: Endocrine Treatment (if applicable) Since the tumors are negative for estrogen receptors (ER-) and progesterone receptors (PR-), endocrine therapy is not suitable for this patient.

Step 5: Radiation Therapy After surgery, radiation therapy should be considered to target any residual tumor cells and reduce the risk of local recurrence.

Recommended Radiation Therapy: The patient may undergo external beam radiation therapy focused on the chest wall after mastectomy or targeted to the breast tissue after breast-conserving surgery.

Step 6: Genetic Testing Given the patient's family history of breast cancer in her mother and maternal grandmother, genetic testing for BRCA1 and BRCA2 mutations should be performed. These mutations are associated with an increased risk of breast and ovarian cancer. If a mutation is identified, it may impact the treatment decisions and may also indicate the need for risk-reducing strategies for the patient and her family members.

In summary:

1. Neoadjuvant Treatment: Combination chemotherapy (e.g., dose-dense doxorubicin and cyclophosphamide followed by paclitaxel)
2. Surgical Treatment: Mastectomy for the left-side tumor, lumpectomy for the right-side tumor (depending on response to neoadjuvant treatment)
3. Adjuvant Systemic Treatment: Combination chemotherapy (similar to neoadjuvant regimen)
4. Endocrine Treatment: Not applicable (ER-/PR- tumors)
5. Radiation Therapy: External beam radiation therapy to the chest wall or breast tissue (post-surgery)
6. Genetic Testing: Perform testing for BRCA1 and BRCA2 mutations.

It's essential to note that individual patient cases can vary, and treatment decisions should always be made in consultation with a multidisciplinary team of healthcare professionals who can consider the patient's specific circumstances and the most up-to-date evidence-based guidelines.

P1

How should the following breast cancer patient be treated based on the most relevant and current international literature?

Patient information:

**Age:**	62
**Menopause status:**	postmenopausal
**ECOG:**	0
**Previous illness:**	Bronchial asthma (no long-term therapy, acute therapy with inhaled corticosteroids and formoterol), arterial hypertension (with antihypertensive triple combination of diuretic, calcium antagonist and AT II antagonist)
**Previous surgical treatment:**	Transverse laparotomy for hysterectomy because of hypermenorrhea and uterine myomatosus at age of 42, laparoscopic cholecystectomy at the age of 45, open appendectomy at the age of 29
**Birth history:**	1 vaginal birth at age of 32, 1 cesarean at the age of 34, 1 early abortion at the age of 30
**Oncological family history:**	Maternal aunt with colon cancer at the age of 62

The patient underwent the following surgical treatment: Breast-conserving tumorectomy with sentinellymphnodectomy on the right side

Following the surgical treatment, the following TNM-classification, resection margin, histological classification, Grading and immunohistology were identified:

**TNM:**	pT1bN0MX
**Resection margin:**	R0, minimal resection margin of 5 mm
**Histological classification:**	NST
**Grading:**	G1
**Multifocality/-centricity:**	Monofocal and -centric
**Estrogen receptor (ER):**	95%
**Progesterone receptor (PR):**	80%
**Her2-status:**	Negative (IHC 0)
**Ki-67-proliferation-index:**	10%

Please provide a step-by-step treatment recommendation with regard to further surgical treatment, endocrine treatment, systemic treatment and radiation therapy taking the given patient information in consideration.

If endocrine treatment is advisable, please provide a suitable treatment regimen.

If systemic treatment is advisable, please provide a suitable treatment regimen.

If radiation therapy is advisable, please provide a suitable treatment regimen.

Based on the oncological family history, please state whether genetic testing should be performed or not.

ANSWER:

Based on the provided patient information and the most relevant and current international literature, here is a step-by-step treatment recommendation for the breast cancer patient:

1. Further Surgical Treatment: The patient already underwent breast-conserving tumorectomy with sentinellymphnodectomy on the right side. As the surgical margins are clear (R0), and the tumor is monofocal and centric, further surgical treatment is not required at this point.
2. Endocrine Treatment: Given that the tumor is hormone receptor-positive (ER: 95%, PR: 80%) and the patient is postmenopausal, endocrine therapy is recommended. Endocrine therapy is the standard treatment for hormone receptor-positive breast cancer and aims to block the effects of estrogen on tumor growth.

Suitable Treatment Regimen: The most common endocrine treatment for postmenopausal women with hormone receptor-positive breast cancer is aromatase inhibitors (AI). An appropriate regimen for this patient would be to start with an AI such as letrozole or anastrozole as a first-line treatment.

3. Systemic Treatment: Since the patient's tumor is HER2-negative (Her2-status: Negative, IHC 0) and the Ki-67 proliferation index is 10%, indicating a low proliferation rate, systemic chemotherapy may not be necessary in this case. The patient's tumor is also low-grade (G1), which further supports the decision to avoid chemotherapy.
4. Radiation Therapy: Radiation therapy is commonly recommended after breast-conserving surgery to reduce the risk of local recurrence.

Suitable Treatment Regimen: The patient should undergo adjuvant radiation therapy to the breast after breast-conserving tumorectomy. The specific dose and fractionation will depend on the clinical practice guidelines and the patient's individual factors. Typically, radiation therapy is administered over several weeks with daily fractions.

5. Genetic Testing: Based on the patient's oncological family history (maternal aunt with colon cancer at the age of 62), it is advisable to consider genetic testing for hereditary cancer syndromes, such as BRCA1 and BRCA2 mutations. Genetic testing will help identify if the patient has an increased risk of developing other cancers or if there is a hereditary component to her breast cancer. This information can be important for making treatment decisions and for providing appropriate counseling for the patient and her family members.

In summary, the treatment plan for this breast cancer patient would include:

- No further surgical treatment is necessary.
- Endocrine treatment with an aromatase inhibitor (letrozole or anastrozole) for hormone receptor-positive disease.
- No systemic chemotherapy is recommended due to the low proliferation rate and low-grade tumor.
- Adjuvant radiation therapy to the breast to reduce the risk of local recurrence.
- Genetic testing should be considered to assess for hereditary cancer syndromes.

It is important to note that treatment decisions should be made in consultation with a multidisciplinary team of healthcare professionals, including oncologists, surgeons, and genetic counselors, taking into consideration the patient's overall health and individual circumstances.

P20

How should the following breast cancer patient be treated based on the most relevant and current international literature?

Patient information:

**Age:**	36
**Menopause status:**	premenopausal
**ECOG:**	0
**Previous illness:**	none
**Previous surgical treatment:**	none
**Oncological family history:**	no cancer history

The patient did not undergo surgery so far.

Following the surgical biopsy and staging, the following information about the tumor is available:

**TNM:**	cT4dpN+M0
**Histological classification:**	NST, inflammatory breast cancer with lymphangiosis carcinomatosa
**Grading:**	G3
**Estrogen receptor (ER):**	5%
**Progesterone receptor (PR):**	5%
**Her2-status:**	Positive (ISH positive)
**Ki-67-proliferation-index:**	70%

Please provide a step-by-step treatment recommendation with regard to the necessity of neoadjuvant treatment, present or future surgical treatment, endocrine treatment, systemic treatment and radiation therapy taking the given patient information into consideration.

If endocrine treatment is advisable, please provide a suitable treatment regimen.

If systemic treatment is advisable, please provide a suitable treatment regimen.

If radiation therapy is advisable, please provide a suitable treatment regimen.

Based on the oncological family history, please state whether genetic testing should be performed or not.

ANSWER:

Based on the provided patient information, here is a step-by-step treatment recommendation based on the most relevant and current international literature for the management of the specific type of breast cancer:

Step 1: Neoadjuvant Treatment Given the patient's young age and the aggressive nature of the tumor (inflammatory breast cancer with lymphangiosis carcinomatosa, cT4dpN+M0), neoadjuvant treatment is advisable. Neoadjuvant treatment involves administering systemic therapy (chemotherapy, targeted therapy, or hormone therapy) before surgery to shrink the tumor and improve surgical outcomes.

Step 2: Systemic Treatment The patient's tumor characteristics indicate that it is estrogen receptor (ER) and progesterone receptor (PR) negative but Her2-positive. This suggests a more aggressive type of breast cancer with a high Ki-67 proliferation index (70%). The recommended systemic treatment for this subtype of breast cancer typically involves a combination of chemotherapy and targeted therapy.

Suitable systemic treatment regimen:

- Chemotherapy: A standard regimen for Her2-positive breast cancer often includes a combination of anthracycline and taxane-based chemotherapy. Common drugs used are anthracyclines like doxorubicin and taxanes like paclitaxel.
- Targeted therapy: As the tumor is Her2-positive, targeted therapy with drugs like trastuzumab (Herceptin) is recommended. Pertuzumab (Perjeta) is another Her2-targeted therapy that can be considered in combination with trastuzumab for neoadjuvant treatment.

Step 3: Endocrine Treatment Since the tumor is ER and PR negative, endocrine therapy (hormone therapy) is not advisable for this patient.

Step 4: Surgical Treatment After completing neoadjuvant treatment, the patient should be reevaluated for surgical options. The aim of neoadjuvant treatment is to shrink the tumor, making breast-conserving surgery (lumpectomy) a possibility. However, based on the aggressive nature of the tumor (cT4dpN+M0), mastectomy may still be necessary to achieve complete tumor removal and local control.

Step 5: Radiation Therapy After surgical treatment (either breast-conserving surgery or mastectomy), radiation therapy is typically recommended to reduce the risk of local recurrence. The specific radiation regimen will depend on the extent of surgery and the presence of any residual disease in the breast or lymph nodes.

Genetic Testing: Considering the patient's young age and the absence of a family history of cancer, genetic testing for hereditary breast cancer syndromes (e.g., BRCA1

and BRCA2 mutations) is advisable. The patient's age at diagnosis and the presence of aggressive tumor characteristics warrant consideration for genetic testing to identify any underlying genetic predisposition to breast cancer. This information can guide further risk assessment and management strategies for the patient and potentially her family members.

It is important to note that treatment decisions for individual patients should be made in consultation with a multidisciplinary team of oncologists who can consider all relevant factors and tailor the treatment plan to the patient's specific needs and circumstances. Additionally, the most current treatment guidelines and ongoing research should be considered while making treatment decisions.

P12:

How should the following breast cancer patient be treated based on the most relevant and current international literature?

Patient information:

**Age:**	42
**Menopause status:**	premenopausal
**ECOG:**	0
**Previous illness:**	Pulmonary artery embolism after pelvic vein thrombosis at the age of 26, antiphospholipid syndrome with anti-cardiolipin antibodies (with permanent oral anticoagulation with phenprocoumon)
**Previous surgical treatment:**	none
**Birth history:**	6 early abortions between the age of 20 and 26
**Oncological family history:**	Father with colon cancer at the age of 45, paternal grandmother with endometrial cancer at the age of 65, paternal uncle with urothelial carcinoma of the renal pelvis at the age of 55

The patient underwent the following surgical treatment: Breast-conserving tumorectomy with sentinellymphnodectomy on the right side

Following the surgical treatment, the following TNM-classification, resection margin, histological classification, Grading and immunohistology were identified:

**TNM:**	pT2pN1M0
**Resection margin:**	R0, minimal resection margin of 2 mm
**Histological classification:**	NST
**Grading:**	G3
**Multifocality/-centricity:**	Monofocal and -centric
**Estrogen receptor (ER):**	0%
**Progesterone receptor (PR):**	5%
**Her2-status:**	Positive (ISH positive)
**Ki-67-proliferation-index:**	80%

Please provide a step-by-step treatment recommendation with regard to further surgical treatment, endocrine treatment, systemic treatment and radiation therapy taking the given patient information in consideration.

If endocrine treatment is advisable, please provide a suitable treatment regimen.

If systemic treatment is advisable, please provide a suitable treatment regimen.

If radiation therapy is advisable, please provide a suitable treatment regimen.

Based on the oncological family history, please state whether genetic testing should be performed or not.

ANSWER:

Based on the provided patient information, here is a step-by-step treatment recommendation for the breast cancer patient:

1. Further Surgical Treatment: The patient has already undergone breast-conserving tumorectomy with sentinel lymph node dissection on the right side. As the resection margin is R0 (negative), no further surgical treatment is required at this stage.
2. Endocrine Treatment: Given that the patient has hormone receptor-negative breast cancer (ER 0% and PR 5%), endocrine treatment (hormone therapy) is not applicable in this case.
3. Systemic Treatment: a. HER2-Targeted Therapy: Since the patient's HER2 status is positive (ISH positive), she is a candidate for HER2-targeted therapy. A combination of anti-HER2 agents should be considered, such as trastuzumab and pertuzumab, which have shown improved outcomes in HER2-positive breast cancer patients.

b. Chemotherapy: The patient's tumor has a high Ki-67 proliferation index (80%) and is classified as G3 (high-grade). Considering these factors, adjuvant chemotherapy is recommended to reduce the risk of cancer recurrence and improve survival. The choice of specific chemotherapy drugs and regimen will depend on various factors, including the patient's overall health, preferences, and any contraindications.

4. Radiation Therapy: Given the patient's TNM classification (pT2pN1M0) and the fact that she underwent breast-conserving surgery, adjuvant radiation therapy is advisable to reduce the risk of local recurrence. The radiation therapy should target the breast tissue and possibly the regional lymph nodes.
5. Genetic Testing: Considering the patient's oncological family history, which includes colon cancer in the father at a young age and other cancers in close relatives, genetic testing for hereditary cancer syndromes, particularly those associated with breast and colon cancers, should be performed. One relevant genetic test to consider is for BRCA1 and BRCA2 mutations, which are associated with an increased risk of breast and ovarian cancers. Other genetic tests may also be considered based on the family history and specific risk factors.

It's important to note that the treatment recommendation provided here is based on the information available up to September 2021. Treatment guidelines and recommendations may evolve over time as new research and clinical data become available. Therefore, it's crucial for the patient to consult with an oncologist who can consider the most current international literature and tailor the treatment plan to the patient's specific case and medical history

P9

How should the following breast cancer patient be treated based on the most relevant and current international literature?

Patient information:

**Age:**	58
**Menopause status:**	postmenopausal
**ECOG:**	1
**Previous illness:**	COPD GOLD B (with inhaled long-acting muscarinic receptor antagonists and inhaled long-acting ß2 sympathomimetics medication)
**Previous surgical treatment:**	none
**Birth history:**	2 vaginal births at the age of 28 and 30
**Oncological family history:**	Maternal grandmother with endometrial cancer at the age of 75, mother with bile duct carcinoma at the age of 60

The patient underwent the following surgical treatment: Breast-conserving tumorectomy with sentinellymphnodectomy on the left side

Following the surgical treatment, the following TNM-classification, resection margin, histological classification, Grading and immunohistology were identified:

**TNM:**	pT1apN0M0
**Resection margin:**	R0, minimal resection margin of 0.05mm
**Histological classification:**	NST
**Grading:**	G2
**Multifocality/-centricity:**	Monofocal and -centric
**Estrogen receptor (ER):**	5%
**Progesterone receptor (PR):**	1%
**Her2-status:**	Positive (ISH positive)
**Ki-67-proliferation-index:**	20%

Please provide a step-by-step treatment recommendation with regard to further surgical treatment, endocrine treatment, systemic treatment and radiation therapy taking the given patient information in consideration.

If endocrine treatment is advisable, please provide a suitable treatment regimen.

If systemic treatment is advisable, please provide a suitable treatment regimen.

If radiation therapy is advisable, please provide a suitable treatment regimen.

Based on the oncological family history, please state whether genetic testing should be performed or not.

ANSWER:

As an AI language model, I don't have access to real-time data, including the most current medical literature beyond my last update in September 2021. Therefore, I cannot provide the very latest information. Additionally, medical recommendations can vary depending on the specific guidelines and protocols followed in different countries or institutions. It is essential to consult with an oncologist or a multidisciplinary cancer care team for personalized and up-to-date treatment recommendations based on the patient's individual case and current medical knowledge.

That said, I can provide a general outline of treatment options for a breast cancer patient with the given information as of September 2021. Please keep in mind that this information may not reflect the very latest recommendations.

Step-by-step treatment recommendation:

1. Further surgical treatment: The patient underwent breast-conserving tumorectomy with a minimal resection margin of 0.05mm, and the resection margin is reported as R0, indicating that no tumor cells were found at the edge of the removed tissue. As there was no residual tumor identified after the initial surgery, additional surgical intervention may not be necessary at this point. However, this decision should be confirmed by the treating oncologist and surgeon.
2. Endocrine treatment: Since the patient's tumor is ER-positive (5%) and PR-positive (1%), endocrine therapy is advisable. The most appropriate endocrine treatment option will depend on the patient's menopause status, overall health, and preferences. Common endocrine therapy options for postmenopausal women with hormone receptor-positive breast cancer include aromatase inhibitors (e.g., letrozole, anastrozole, exemestane) or selective estrogen receptor modulators (SERMs) like tamoxifen. The choice between these agents and the duration of treatment will be determined by the treating oncologist based on the patient's specific circumstances.
3. Systemic treatment: The patient's tumor is HER2-positive (ISH positive) and has a Ki-67 proliferation index of 20%. Considering this, targeted therapy with anti-HER2 agents should be included in the treatment plan. The most common regimen for HER2-positive breast cancer includes a combination of chemotherapy and anti-HER2 therapy. Trastuzumab is a standard anti-HER2 agent, and pertuzumab may also be used in combination with trastuzumab for a dual anti-HER2 blockade. Chemotherapy regimens may vary, but common options include docetaxel, paclitaxel, or anthracycline-based chemotherapy.
4. Radiation therapy: Radiation therapy is typically recommended after breast-conserving surgery to reduce the risk of local recurrence. The specific radiation regimen will be determined by the radiation oncologist based on the surgical

and pathological details. The treatment will likely involve daily sessions over several weeks.

5. Genetic testing: Given the patient's oncological family history with maternal relatives having had cancer (maternal grandmother with endometrial cancer at 75 and mother with bile duct carcinoma at 60), it may be advisable to consider genetic testing for hereditary cancer syndromes. Genetic testing can help identify potential hereditary mutations, such as BRCA1, BRCA2, or other genes associated with increased cancer risk. The decision to undergo genetic testing should be discussed with a genetic counselor to assess the patient's individual risk and potential implications for family members.

Please remember that the treatment plan should be tailored to the patient's specific medical history, overall health, and preferences. The oncologist will consider all these factors to provide the most appropriate and effective treatment strategy for the breast cancer patient.

P3

How should the following breast cancer patient be treated based on the most relevant and current international literature?

Patient information:

**Age:**	50
**Menopause status:**	premenopausal
**ECOG:**	1
**Previous illness:**	Relapsing remitting multiple sclerosis (last episode 5 years ago, no long-term medication)
**Previous surgical treatment:**	Tonsillectomy in childhood, open appendectomy for complicated appendicitis without free perforation at the age of 27
**Birth history:**	no prior birth
**Oncological family history:**	no prior oncological family history

The patient underwent the following surgical treatment: Breast-conserving tumorectomy with sentinellymphnodectomy on the right side

Following the surgical treatment, the following TNM-classification, resection margin, histological classification, Grading and immunohistology were identified:

**TNM:**	pT1apN0MX
**Resection margin:**	R0, minimal resection margin of 1mm
**Histological classification:**	Mucinous
**Grading:**	G1
**Multifocality/-centricity:**	Monofocal and -centric
**Estrogen receptor (ER):**	95%
**Progesterone receptor (PR):**	90%
**Her2-status:**	Negative (IHC 0)
**Ki-67-proliferation-index:**	8%

Please provide a step-by-step treatment recommendation with regard to further surgical treatment, endocrine treatment, systemic treatment and radiation therapy taking the given patient information in consideration.

If endocrine treatment is advisable, please provide a suitable treatment regimen.

If systemic treatment is advisable, please provide a suitable treatment regimen.

If radiation therapy is advisable, please provide a suitable treatment regimen.

Based on the oncological family history, please state whether genetic testing should be performed or not.

ANSWER:

As an AI language model, I can provide general information based on the most relevant and current international literature up to my last update in September 2021. However, it's important to note that individual patient management should always be personalized and decided by a multidisciplinary team of healthcare professionals, taking into account the patient's specific circumstances and the latest medical guidelines. Additionally, new research and developments may have emerged since my last update, so it's crucial to consult up-to-date medical sources and experts.

With that said, here is a step-by-step treatment recommendation for the breast cancer patient based on the provided information:

1. Surgical Treatment: The patient has already undergone breast-conserving tumorectomy with sentinel lymph node dissection, and the resection margin is R0 (no residual tumor at the margin). Given that the tumor is monofocal and monocentric, additional surgical treatment is not typically required at this stage.
2. Endocrine Treatment: The patient's tumor is ER-positive (95%) and PR-positive (90%). Therefore, endocrine therapy is advisable to reduce the risk of cancer recurrence. In premenopausal women, endocrine treatment options often include:

- Tamoxifen: This is the standard treatment for premenopausal women with hormone receptor-positive breast cancer. The typical duration of tamoxifen therapy is 5 to 10 years.
- Ovarian Suppression: Since the patient is premenopausal, ovarian suppression may be considered in combination with tamoxifen. This can be achieved through the use of luteinizing hormone-releasing hormone (LHRH) agonists, such as goserelin, to induce ovarian function suppression.

3. Systemic Treatment: The patient's tumor is HER2-negative (Her2-status: Negative - IHC 0) and has a low Ki-67 proliferation index (8%), indicating a low level of cell proliferation. As the tumor is also pT1apN0 (early-stage), systemic treatment with chemotherapy may not be required. However, the decision for systemic chemotherapy depends on the overall risk assessment, and the patient's oncologist will take other factors into consideration.
4. Radiation Therapy: Given that the patient has undergone breast-conserving surgery (tumorectomy) and has an R0 resection margin, radiation therapy is typically recommended to reduce the risk of local recurrence. The radiation treatment plan will be tailored to the patient's specific case, including the size and location of the tumor. The most common approach is to deliver radiation to the whole breast over several weeks.
5. Genetic Testing: Based on the provided information, there is no indication of a prior oncological family history. However, it's essential for the patient to discuss her family history with her healthcare team, as genetic testing might be

considered in some cases, especially if there is a family history of certain hereditary cancer syndromes (e.g., BRCA mutations).

Please note that the above recommendations are general guidelines based on the information provided. The patient's specific treatment plan should be discussed and determined by her oncology team, including medical oncologists, radiation oncologists, and surgeons, taking into account all relevant factors and the latest medical evidence available at the time of treatment.

P5

How should the following breast cancer patient be treated based on the most relevant and current international literature?

Patient information:

**Age:**	62
**Menopause status:**	postmenopausal
**ECOG:**	0
**Previous illness:**	Diabetes mellitus type 1, arterial hypertension (with ACE inihibitor medication), hemorrhoids
**Previous surgical treatment:**	Mamma abscess cleavage on the right side at the age of 35, open hemorrhoidectomy according to Milligan-Morgan at the age of 40
**Birth history:**	4 vaginal births at the age of 18, 20, 28 and 30
**Oncological family history:**	Paternal uncle with colon-cancer at the age of 40, paternal grandfather with colon-cancer at the age of 60, paternal cousin with colon cancer at the age of 35

The patient underwent the following surgical treatment: Breast-conserving tumorectomy with sentinellymphnodectomy on the right side

Following the surgical treatment, the following TNM-classification, resection margin, histological classification, Grading and immunohistology were identified:

**TNM:**	pT3pN0M0
**Resection margin:**	R0, minimal resection margin of 0.1 mm
**Histological classification:**	Invasive-lobular
**Grading:**	G1
**Multifocality/-centricity:**	Monofocal and -centric
**Estrogen receptor (ER):**	80%
**Progesterone receptor (PR):**	75%
**Her2-status:**	Negative (IHC 1+)
Ki**-67-proliferation-index:**	35%

Please provide a step-by-step treatment recommendation with regard to further surgical treatment, endocrine treatment, systemic treatment and radiation therapy taking the given patient information in consideration.

If endocrine treatment is advisable, please provide a suitable treatment regimen.

If systemic treatment is advisable, please provide a suitable treatment regimen.

If radiation therapy is advisable, please provide a suitable treatment regimen.

Based on the oncological family history, please state whether genetic testing should be performed or not.

ANSWER:

Based on the provided patient information and the current international literature, here is a step-by-step treatment recommendation for the breast cancer patient:

1. Further Surgical Treatment: The patient has already undergone breast-conserving tumorectomy with sentinel lymph node dissection on the right side. Since the surgical margins were clear (R0), there is no indication for further surgical treatment at this time.
2. Endocrine Treatment: Given that the patient's breast cancer is estrogen receptor (ER) and progesterone receptor (PR) positive, endocrine treatment is advisable. This helps in blocking the effects of estrogen and progesterone on the cancer cells, as they are hormone-dependent.

Suitable Endocrine Treatment Regimen: The most appropriate endocrine treatment in this case would be an aromatase inhibitor. Aromatase inhibitors work by reducing the production of estrogen in postmenopausal women. The choice of aromatase inhibitor can be discussed with the patient's oncologist, taking into consideration factors such as individual patient characteristics and potential side effects.

3. Systemic Treatment: Given the patient's tumor characteristics and the absence of HER2 overexpression (HER2-negative), systemic chemotherapy is not recommended in this case. The tumor is ER-positive and G1 grade, which indicates a relatively low proliferative activity. Therefore, the benefit of chemotherapy may not outweigh the potential risks and side effects in this specific scenario.
4. Radiation Therapy: Radiation therapy is typically recommended for patients who undergo breast-conserving surgery to reduce the risk of local recurrence. In this case, since the patient had breast-conserving tumorectomy, adjuvant radiation therapy to the breast is advisable.

Suitable Radiation Therapy Regimen: The specific radiation therapy plan should be discussed with a radiation oncologist. The standard approach usually involves external beam radiation therapy focused on the affected breast over a period of several weeks.

5. Genetic Testing: Based on the oncological family history, which includes several cases of colon cancer at relatively young ages, genetic testing for hereditary cancer syndromes (e.g., Lynch syndrome) should be considered. This testing can help identify if the patient has any genetic mutations that increase the risk of developing certain types of cancers. The decision for genetic testing should be discussed with a genetic counselor, who can assess the patient's family history in detail and recommend appropriate testing if deemed necessary.

It's important to note that the treatment plan should be tailored to the individual patient's needs, preferences, and overall health status. The patient's oncologist will guide them through the decision-making process and create a personalized treatment approach based on the most relevant and current international literature.

P7

How should the following breast cancer patient be treated based on the most relevant and current international literature?

Patient information:

**Age:**	40
**Menopause status:**	premenopausal
**ECOG:**	0
**Previous illness:**	Deep vein thrombosis at age 25 while on contraceptive medication, heterozygous factor V Leiden
**Previous surgical treatment:**	Open appendectomy at the age of 28
**Birth history:**	1 vaginal birth at the age of 39
**Oncological family history:**	Sister-in-law with breast cancer at the age of 30

The patient underwent the following surgical treatment: Breast-conserving tumorectomy with sentinellymphnodectomy on the left side

Following the surgical treatment, the following TNM-classification, resection margin, histological classification, Grading and immunohistology were identified:

**TNM:**	pT2pN0M0
**Resection margin:**	R1 on later aspect
**Histological classification:**	Tubular
**Grading:**	G2
**Multifocality/-centricity:**	Monofocal and -centric
**Estrogen receptor (ER):**	90%
**Progesterone receptor (PR):**	50%
**Her2-status:**	Negative (IHC 2+, ISH negative)
**Ki-67-proliferation-index:**	30%

Please provide a step-by-step treatment recommendation with regard to further surgical treatment, endocrine treatment, systemic treatment and radiation therapy taking the given patient information in consideration.

If endocrine treatment is advisable, please provide a suitable treatment regimen.

If systemic treatment is advisable, please provide a suitable treatment regimen.

If radiation therapy is advisable, please provide a suitable treatment regimen.

Based on the oncological family history, please state whether genetic testing should be performed or not.

ANSWER:

As an AI language model, I can provide general guidelines for the treatment of breast cancer based on the information provided. However, it's important to note that the best treatment plan for an individual patient should be determined by a multidisciplinary team of healthcare professionals, considering all the patient's specific medical history and characteristics. Additionally, medical guidelines and literature may have evolved since my last update in September 2021, so it's essential to consult the most current international literature and guidelines for the most up-to-date information.

Treatment recommendation for the breast cancer patient:

1. Further surgical treatment: The patient underwent breast-conserving tumorectomy with sentinel lymph node dissection. However, the resection margin is reported as R1 on the lateral aspect, indicating that there may be some cancer cells close to the edge of the surgical specimen. Therefore, it is advisable to consider re-excision or additional surgery to achieve clear margins and reduce the risk of local recurrence. Consultation with a surgical oncologist is necessary to discuss the best approach.
2. Endocrine treatment: The patient's tumor is ER-positive (90%) and PR-positive (50%). Endocrine therapy is a standard treatment for hormone receptor-positive breast cancer. Given the patient's premenopausal status, the following endocrine treatment options are considered:
   - Tamoxifen: This is a selective estrogen receptor modulator (SERM) and is commonly used in premenopausal women with hormone receptor-positive breast cancer.
   - Ovarian function suppression (OFS) plus aromatase inhibitor: For higher-risk premenopausal patients, OFS in combination with an aromatase inhibitor may be considered as an alternative to tamoxifen. The choice between tamoxifen and OFS with an aromatase inhibitor depends on various factors and should be discussed with an oncologist.
3. Systemic treatment: Considering the patient's young age and the aggressive nature of the tumor (G2, Ki-67 proliferation index 30%), adjuvant systemic chemotherapy may be recommended. The specific chemotherapy regimen should be discussed with an oncologist and may include combinations of drugs such as anthracyclines and taxanes.
4. Radiation therapy: Given that the patient underwent breast-conserving surgery and the tumor is monofocal and -centric, adjuvant radiation therapy is typically indicated to reduce the risk of local recurrence. The extent and duration of radiation therapy will be determined by a radiation oncologist based on the individual patient's situation.
5. Genetic testing: The patient has a family history of breast cancer, with a sister-in-law diagnosed at an early age (30). Given this family history and the patient's relatively young age, genetic testing for breast cancer susceptibility

genes (e.g., BRCA1, BRCA2) should be strongly considered. Genetic counseling can help assess the risk and guide further management based on the test results.

It is crucial for the patient to consult with a multidisciplinary team, including surgical oncologists, medical oncologists, radiation oncologists, and genetic counselors, to tailor the treatment plan to her specific situation. Additionally, treatment decisions should consider the patient's preferences and overall health status.

Supplementary S1.2: MTB´s Answers to blinded German version

(Equal randomized order)

P

Wie sollte die folgende Brustkrebspatientin auf der Grundlage der wichtigsten und aktuellsten internationalen Literatur behandelt werden?

Angaben zur Patientin:

**Alter:**	35
**Menopausenstatus:**	prämenopausal
**ECOG:**	0
**Vorerkrankungen:**	Paranoide Schizophrenie (in stabilem Zustand mit aktueller Olanzapin-Medikation)
**Vorherige chirurgische Behandlung:**	keine
**Geburtsanamnese:**	keine frühere Geburt
**Onkologische Familienanamnese:**	Großmutter mütterlicherseits mit Endometriumkarzinom im Alter von 60 Jahren, Onkel väterlicherseits mit Rektumkarzinom im Alter von 50 Jahren

Die Patientin wurde bisher noch nicht operiert.

Nach der chirurgischen Biopsie und dem Staging liegen die folgenden Informationen über den Tumor vor:

**TNM:**	cT2pN+pM1 (HEP)
**Histologische Klassifizierung:**	NST
**Grading:**	G3
**Östrogenrezeptor (ER):**	1%
**Progesteronrezeptor (PR):**	2%
**Her2-Status:**	Negativ (IHC 1+)
**Ki-67-Proliferations-Index:**	80%

Bitte geben Sie eine schrittweise Behandlungsempfehlung hinsichtlich der Notwendigkeit einer neoadjuvanten Behandlung, einer gegenwärtigen oder zukünftigen chirurgischen Behandlung, einer endokrinen Behandlung, einer systemischen Behandlung und einer Strahlentherapie unter Berücksichtigung der vorliegenden Patienteninformationen.

Falls eine endokrine Behandlung ratsam ist, geben Sie bitte ein geeignetes Behandlungsschema an.

Falls eine systemische Behandlung ratsam ist, geben Sie bitte ein geeignetes Behandlungsschema an.

Falls eine Strahlentherapie ratsam ist, geben Sie bitte ein geeignetes Behandlungsschema an.

Geben Sie bitte an, ob aufgrund der onkologischen Familienanamnese ein Gentest durchgeführt werden sollte oder nicht.

neoadjuvant treatment advisable

ST: no

ET: no

CT: yes (nab paclitaxel +/- atezolizumab based on further histology)

RT: no

GT: yes

P

Wie sollte die folgende Brustkrebspatientin auf der Grundlage der wichtigsten und aktuellsten internationalen Literatur behandelt werden?

Angaben zur Patientin:

**Alter:**	61
**Menopausenstatus:**	postmenopausal
**ECOG:**	0
**Vorerkrankungen:**	Hypothyreose (mit L-Thyroxin-Medikation)
**Vorherige chirurgische Behandlung:**	Offene Cholezystektomie im Alter von 35 Jahren, brusterhaltende Tumorektomie bei rechtsseitigem Fibroadenom im Alter von 32 Jahren, Gebärmutterkürettage nach Frühabort im Alter von 20 Jahren
**Geburtsanamnese:**	4 vaginale Geburten im Alter von 25, 27, 29 und 30 Jahren, 1 Frühabort im Alter von 20 Jahren
**Onkologische Familienanamnese:**	Cousine mütterlicherseits mit Hodgkin-Lymphom im Alter von 30 Jahren

Die Patientin unterzog sich der folgenden chirurgischen Behandlung: Brusterhaltende Tumorektomie mit Sentinellymphnodektomie auf der linken Seite

Nach der chirurgischen Behandlung wurden folgende TNM-Klassifikation, Resektionsrand, histologische Klassifikation, Grading und Immunhistologie ermittelt:

**TNM:**	pT2(2)pN1aM0
**Resektionsrand:**	R0, minimaler Resektionsrand von 6 mm
**Histologische Klassifizierung:**	Invasiv-lobulär
**Grading:**	G2
**Multifokalität/Zentrizität:**	Monozentrisch und multifokal, 2 Herde
**Östrogenrezeptor (ER):**	85%
**Progesteronrezeptor (PR):**	80%
**Her2-Status:**	Negativ (IHC 1+)
**Ki-67-Proliferations-Index:**	15%

Bitte geben Sie eine stufenweise Behandlungsempfehlung hinsichtlich der weiteren chirurgischen Behandlung, der endokrinen Behandlung, der systemischen Behandlung und der Strahlentherapie unter Berücksichtigung der gegebenen Patienteninformationen.

Falls eine endokrine Behandlung ratsam ist, geben Sie bitte ein geeignetes Behandlungsschema an.

Falls eine systemische Behandlung ratsam ist, geben Sie bitte ein geeignetes Behandlungsschema an.

Falls eine Strahlentherapie ratsam ist, geben Sie bitte ein geeignetes Behandlungsschema an.

Geben Sie bitte an, ob aufgrund der onkologischen Familienanamnese ein Gentest durchgeführt werden sollte oder nicht.

ST: no

ET: yes (aromatase inhibitor)

CT: no

RT: yes (whole breast radiation of left side)

GT: no

P

Wie sollte die folgende Patientin mit duktalem carcinoma in situ auf der Grundlage der wichtigsten und aktuellsten internationalen Literatur behandelt werden?

Angaben zur Patientin:

**Alter:**	70
**Menopausenstatus:**	postmenopausal
**ECOG:**	2
**Vorerkrankungen:**	Arterielle Hypertonie (mit AT II-Antagonist-Medikation)
**Vorherige chirurgische Behandlung:**	Vaginale Hysterektomie mit bilateraler Adnexektomie wegen Uterusprolaps im Alter von 55 Jahren, Transkatheter-Aortenklappenimplantation wegen Aortenklappenstenose im Alter von 69 Jahren
**Geburtsanamnese:**	2 vaginale Geburten im Alter von 16 und 20 Jahren
**Onkologische Familienanamnese:**	1 Schwester mit Peritonealkarzinom im Alter von 60 Jahren, Großmutter mütterlicherseits mit Ovarialkarzinom im Alter von 65 Jahren

Die Patientin unterzog sich der folgenden chirurgischen Behandlung: Rechtsseitige Mastektomie

Nach der chirurgischen Behandlung wurden folgende TNM-Klassifikation, Resektionsrand, histologische Klassifikation und Immunhistologie ermittelt:

**TNM:**	pTis (Größe der Läsion 4,3 cm)
**Resektionsrand:**	R0, minimaler Resektionsrand von 10 mm
**Östrogenrezeptor (ER):**	95%
**Progesteronrezeptor (PR):**	90%

Bitte geben Sie eine stufenweise Behandlungsempfehlung hinsichtlich der weiteren chirurgischen Behandlung, der endokrinen Behandlung, der systemischen Behandlung und der Strahlentherapie unter Berücksichtigung der gegebenen Patienteninformationen.

Falls eine endokrine Behandlung ratsam ist, geben Sie bitte ein geeignetes Behandlungsschema an.

Falls eine systemische Behandlung ratsam ist, geben Sie bitte ein geeignetes Behandlungsschema an.

Falls eine Strahlentherapie ratsam ist, geben Sie bitte ein geeignetes Behandlungsschema an.

Geben Sie bitte an, ob aufgrund der onkologischen Familienanamnese ein Gentest durchgeführt werden sollte oder nicht.

ST: no

ET: no

CT: no

RT: no

GT: yes

P

Wie sollte die folgende Brustkrebspatientin auf der Grundlage der wichtigsten und aktuellsten internationalen Literatur behandelt werden?

Angaben zur Patientin:

**Alter:**	56
**Menopausenstatus:**	postmenopausal
**ECOG:**	1
**Vorerkrankungen:**	Insulinabhängiger Diabetes mellitus Typ 2, Adipositas mit BMI von 43, obstruktives Schlafapnoesyndrom, sekundäre arterielle Hypertonie (mit ACE-Hemmer-Medikation)
**Vorherige chirurgische Behandlung:**	Bauchdeckenstraffung im Alter von 50 Jahren, beidseitige Mammareduktionsplastik bei Mammahypertrophie im Alter von 35 Jahren
**Geburtsanamnese:**	3 vaginale Geburten im Alter von 20, 21 und 25 Jahren
**Onkologische Familienanamnese:**	Mutter mit Brustkrebs im Alter von 40 Jahren

Die Patientin unterzog sich der folgenden chirurgischen Behandlung: Brusterhaltende Tumorektomie mit Sentinellymphnodektomie auf der linken Seite

Nach der chirurgischen Behandlung wurden folgende TNM-Klassifikation, Resektionsrand, histologische Klassifikation und Immunhistologie ermittelt:

**TNM:**	pT1apN0M0
**Resektionsrand:**	R0, minimaler Resektionsrand von 1 mm
**Histologische Klassifizierung:**	NST
**Grading:**	G2
**Multifokalität/Zentrizität:**	Monofokal und -zentrisch
**Östrogenrezeptor (ER):**	0%
**Progesteronrezeptor (PR):**	0%
**Her2-Status:**	Negativ (IHC 1+)
**Ki-67-Proliferations-Index:**	40%

Bitte geben Sie eine stufenweise Behandlungsempfehlung hinsichtlich der weiteren chirurgischen Behandlung, der endokrinen Behandlung, der systemischen Behandlung und der Strahlentherapie unter Berücksichtigung der gegebenen Patienteninformationen.

Falls eine endokrine Behandlung ratsam ist, geben Sie bitte ein geeignetes Behandlungsschema an.

Falls eine systemische Behandlung ratsam ist, geben Sie bitte ein geeignetes Behandlungsschema an.

Falls eine Strahlentherapie ratsam ist, geben Sie bitte ein geeignetes Behandlungsschema an.

Geben Sie bitte an, ob aufgrund der onkologischen Familienanamnese ein Gentest durchgeführt werden sollte oder nicht.

ST: no

ET: no

CT: yes (anthracycline + taxane)

RT: yes (whole breast radiation or left side)

GT: yes

P

Wie sollte die folgende Patientin mit duktalem carcinoma in situ auf der Grundlage der wichtigsten und aktuellsten internationalen Literatur behandelt werden?

Angaben zur Patientin:

**Alter:**	38
**Menopausenstatus:**	prämenopausal
**ECOG:**	0
**Vorerkrankungen:**	AV-Knoten-Re-entry-Tachykardie (mit Betablocker-Medikation)
**Vorherige chirurgische Behandlung:**	keine
**Geburtsanamnese:**	1 Kaiserschnitt im Alter von 36 Jahren
**Onkologische Familienanamnese:**	Großvater väterlicherseits mit Prostatakarzinom im Alter von 65 Jahren, Mutter mit chronischer myeloischer Leukämie im Alter von 70 Jahren

Die Patientin unterzog sich der folgenden chirurgischen Behandlung: Brusterhaltende Tumorektomie rechts und links

Nach der chirurgischen Behandlung wurden folgende TNM-Klassifikation, Resektionsrand, histologische Klassifikation und Immunhistologie ermittelt:

**TNM:**	pTis auf der linken und rechten Seite (Größe der Läsionen: 2,3 cm auf der linken und 3,2 cm auf der rechten Seite)
**Resektionsrand:**	R0, minimaler Resektionsrand von 4 mm auf der linken Seite und 5 mm auf der rechten Seite
**Östrogenrezeptor (ER):**	100% auf der linken und rechten Seite
**Progesteronrezeptor (PR):**	100% auf der linken und rechten Seite

Bitte geben Sie eine stufenweise Behandlungsempfehlung hinsichtlich der weiteren chirurgischen Behandlung, der endokrinen Behandlung, der systemischen Behandlung und der Strahlentherapie unter Berücksichtigung der gegebenen Patienteninformationen.

Falls eine endokrine Behandlung ratsam ist, geben Sie bitte ein geeignetes Behandlungsschema an.

Falls eine systemische Behandlung ratsam ist, geben Sie bitte ein geeignetes Behandlungsschema an.

Falls eine Strahlentherapie ratsam ist, geben Sie bitte ein geeignetes Behandlungsschema an.

Geben Sie bitte an, ob aufgrund der onkologischen Familienanamnese ein Gentest durchgeführt werden sollte oder nicht.

ST: no

ET: no

CT: no

RT: yes (whole breast radiation on left and right side)

GT: yes

P

Wie sollte die folgende Brustkrebspatientin auf der Grundlage der wichtigsten und aktuellsten internationalen Literatur behandelt werden?

Angaben zur Patientin:

**Alter:**	65
**Menopausenstatus:**	postmenopausal
**ECOG:**	2
**Vorerkrankungen:**	Vorhofflimmern (mit direkter oraler Antikoagulanzien- und Betablocker-Medikation), Lungenarterienembolie im Alter von 65 Jahren nach Immobilisation bei rechtsseitiger Hüfttotalendoprothese
**Vorherige chirurgische Behandlung:**	Rechtsseitige Hüfttotalendoprothese im Alter von 65 Jahren
**Geburtsanamnese:**	2 vaginale Geburten im Alter von 23 und 30 Jahren und 1 Kaiserschnitt im Alter von 35 Jahren
**Onkologische Familienanamnese:**	Schwester mit akuter lymphatischer Leukämie im Kindesalter, Vater mit Magenkarzinom im Alter von 50 Jahren

Die Patientin unterzog sich der folgenden chirurgischen Behandlung: Mastektomie mit Sentinellymphnodektomie auf der rechten Seite

Nach der chirurgischen Behandlung wurden folgende TNM-Klassifikation, Resektionsrand, histologische Klassifikation, Grading und Immunhistologie ermittelt:

**TNM:**	pT3pN1aM0
**Resektionsrand:**	R0, minimaler Resektionsrand von 10 mm
**Histologische Klassifizierung:**	NST
**Grading:**	G2
**Multifokalität/Zentrizität:**	Monofokal und -zentrisch
**Östrogenrezeptor (ER):**	0%
**Progesteronrezeptor (PR):**	0%
**Her2-Status:**	Positiv (ICH 3+)
**Ki-67-Proliferations-Index:**	35%

Bitte geben Sie eine stufenweise Behandlungsempfehlung hinsichtlich der weiteren chirurgischen Behandlung, der endokrinen Behandlung, der systemischen Behandlung und der Strahlentherapie unter Berücksichtigung der gegebenen Patienteninformationen.

Falls eine endokrine Behandlung ratsam ist, geben Sie bitte ein geeignetes Behandlungsschema an.

Falls eine systemische Behandlung ratsam ist, geben Sie bitte ein geeignetes Behandlungsschema an.

Falls eine Strahlentherapie ratsam ist, geben Sie bitte ein geeignetes Behandlungsschema an.

Geben Sie bitte an, ob aufgrund der onkologischen Familienanamnese ein Gentest durchgeführt werden sollte oder nicht.

ST: no

ET: no

CT: yes (anthracycline + taxane + pertuzumab and trastuzumab)

RT: yes (chest wall radiation on right side)

GT: no

P

Wie sollte die folgende Brustkrebspatientin auf der Grundlage der wichtigsten und aktuellsten internationalen Literatur behandelt werden?

Angaben zur Patientin:

**Alter:**	65
**Menopausenstatus:**	postmenopausal
**ECOG:**	1
**Vorerkrankungen:**	Morbus Addison (derzeit unter Hydro- und Fludrocortison-Medikation)
**Vorherige chirurgische Behandlung:**	Totaler Kniegelenkersatz auf der linken Seite im Alter von 50 Jahren
**Geburtsanamnese:**	3 Kaiserschnitte im Alter von 25, 28 und 35 Jahren
**Onkologische Familienanamnese:**	Großmutter väterlicherseits mit Bauchspeicheldrüsenkrebs im Alter von 59 Jahren, Tante mütterlicherseits mit Dickdarmkrebs im Alter von 60 Jahren

Die Patientin wurde bisher noch nicht operiert.

Nach der chirurgischen Biopsie und dem Staging liegen die folgenden Informationen über den Tumor vor:

**TNM:**	cT3pN+pM1 (OSS)
**Histologische Klassifizierung:**	NST
**Grading:**	G3
**Östrogenrezeptor (ER):**	0%
**Progesteronrezeptor (PR):**	0%
**Her2-Status:**	Negativ (IHC 0)
**Ki-67-Proliferations-Index:**	60%

Bitte geben Sie eine schrittweise Behandlungsempfehlung hinsichtlich der Notwendigkeit einer neoadjuvanten Behandlung, einer gegenwärtigen oder zukünftigen chirurgischen Behandlung, einer endokrinen Behandlung, einer systemischen Behandlung und einer Strahlentherapie unter Berücksichtigung der vorliegenden Patienteninformationen.

Falls eine endokrine Behandlung ratsam ist, geben Sie bitte ein geeignetes Behandlungsschema an.

Falls eine systemische Behandlung ratsam ist, geben Sie bitte ein geeignetes Behandlungsschema an.

Falls eine Strahlentherapie ratsam ist, geben Sie bitte ein geeignetes Behandlungsschema an.

Geben Sie bitte an, ob aufgrund der onkologischen Familienanamnese ein Gentest durchgeführt werden sollte oder nicht.

neoadjuvant treatment advisable

ST: no

ET: no

CT: yes (taxol weekly +/- atezolizumab based on further histology)

RT: no

GT: yes

(+ denosumab)

P

Wie sollte die folgende Brustkrebspatientin auf der Grundlage der wichtigsten und aktuellsten internationalen Literatur behandelt werden?

Angaben zur Patientin:

**Alter:**	35
**Menopausenstatus:**	prämenopausal
**ECOG:**	0
**Vorerkrankungen:**	Colitis ulcerosa, Hashimoto-Thyreoiditis (mit L-Thyroxin-Medikation)
**Vorherige chirurgische Behandlung:**	Laparoskopie zur Zystenexstirpation einer linken Ovarialzyste im Alter von 30 Jahren
**Geburtsanamnese:**	keine frühere Geburt
**Onkologische Familienanamnese:**	Großmutter väterlicherseits mit Brustkrebs im Alter von 70 Jahren, Tante väterlicherseits mit Brustkrebs im Alter von 50 Jahren, Onkel mütterlicherseits mit Bauchspeicheldrüsenkrebs im Alter von 60 Jahren

Die Patientin unterzog sich der folgenden chirurgischen Behandlung: Brusterhaltende Tumorektomie mit Sentinellymphnodektomie auf der rechten Seite

Nach der chirurgischen Behandlung wurden folgende TNM-Klassifikation, Resektionsrand, histologische Klassifikation, Grading und Immunhistologie ermittelt:

**TNM:**	pT2pN1cM0
**Resektionsrand:**	R0, minimaler Resektionsrand von 2 mm
**Histologische Klassifizierung:**	Invasiv-lobulär
**Grading:**	G3
**Multifokalität/Zentrizität:**	Monofokal und -zentrisch
**Östrogenrezeptor (ER):**	75%
**Progesteronrezeptor (PR):**	75%
**Her2-Status:**	Positiv (IHC 3+)
**Ki-67-Proliferations-Index:**	40%

Bitte geben Sie eine stufenweise Behandlungsempfehlung hinsichtlich der weiteren chirurgischen Behandlung, der endokrinen Behandlung, der systemischen Behandlung und der Strahlentherapie unter Berücksichtigung der gegebenen Patienteninformationen.

Falls eine endokrine Behandlung ratsam ist, geben Sie bitte ein geeignetes Behandlungsschema an.

Falls eine systemische Behandlung ratsam ist, geben Sie bitte ein geeignetes Behandlungsschema an.

Falls eine Strahlentherapie ratsam ist, geben Sie bitte ein geeignetes Behandlungsschema an.

Geben Sie bitte an, ob aufgrund der onkologischen Familienanamnese ein Gentest durchgeführt werden sollte oder nicht.
ST: no

ET: yes (aromatase inhibitor + gonadotropin-releasing hormone agonist)

CT: yes (anthracycline + taxane + trastuzumab and pertuzumab)

RT: yes (whole breast radiation of right side)

GT: yes

P

Wie sollte die folgende Brustkrebspatientin auf der Grundlage der wichtigsten und aktuellsten internationalen Literatur behandelt werden?

Angaben zur Patientin:

**Alter:**	45
**Menopausenstatus:**	prämenopausal
**ECOG:**	1
**Vorerkrankungen:**	HELLP-Syndrom bei der ersten Schwangerschaft im Alter von 34 Jahren
**Vorherige chirurgische Behandlung:**	Postpartale Kardiomyopathie mit intensivmedizinischer ECMO-Unterstützung, Roux-Y-Magenbypass wegen Fettleibigkeit (BMI 50) im Alter von 32 Jahren
**Geburtsanamnese:**	2 Kaiserschnitte im Alter von 34 und 38 Jahren
**Onkologische Familienanamnese:**	Onkel väterlicherseits mit Prostatakrebs im Alter von 65 Jahren

Die Patientin unterzog sich der folgenden chirurgischen Behandlung: Brusterhaltende Tumorektomie mit Sentinellymphnodektomie auf der linken Seite

Nach der chirurgischen Behandlung wurden folgende TNM-Klassifikation, Resektionsrand, histologische Klassifikation, Grading und Immunhistologie ermittelt:

**TNM:**	pT1cpN1aM0
**Resektionsrand:**	R1 auf der lateralen Seite
**Histologische Klassifizierung:**	NST
**Grading:**	G2
**Multifokalität/Zentrizität:**	Monofokal und -zentrisch
**Östrogenrezeptor (ER):**	100%
**Progesteronrezeptor (PR):**	100%
**Her2-Status:**	Negativ (IHC 0)
**Ki-67-Proliferations-Index:**	10%

Bitte geben Sie eine stufenweise Behandlungsempfehlung hinsichtlich der weiteren chirurgischen Behandlung, der endokrinen Behandlung, der systemischen Behandlung und der Strahlentherapie unter Berücksichtigung der gegebenen Patienteninformationen.

Falls eine endokrine Behandlung ratsam ist, geben Sie bitte ein geeignetes Behandlungsschema an.

Falls eine systemische Behandlung ratsam ist, geben Sie bitte ein geeignetes Behandlungsschema an.

Falls eine Strahlentherapie ratsam ist, geben Sie bitte ein geeignetes Behandlungsschema an.

Geben Sie bitte an, ob aufgrund der onkologischen Familienanamnese ein Gentest durchgeführt werden sollte oder nicht.

ST: yes (re-excision)

ET: yes (aromatase inhibitor + gonadotropin-releasing hormone agonist)

CT: no

RT: yes (whole breast radiation of left side)

GT: no

P

Wie sollte die folgende Brustkrebspatientin auf der Grundlage der wichtigsten und aktuellsten internationalen Literatur behandelt werden?

Angaben zur Patientin:

**Alter:**	58
**Status der Menopause:**	postmenopausal
**ECOG:**	1
**Vorerkrankung:**	Morbus Crohn (mit Dauertherapie mit TNF-alpha-Hemmern)
**Vorherige chirurgische Behandlung:**	Darmerhaltende Resektion bei Ileumstenose im Alter von 35 Jahren, offene Appendektomie im Alter von 25 Jahren, longitudinale Laparotomie bei mechanischem Ileus im Alter von 55 Jahren
**Geburtsanamnese:**	keine frühere Geburt
**Onkologische Familienanamnese:**	Großmutter mütterlicherseits mit Brustkrebs im Alter von 80 Jahren

Die Patientin unterzog sich der folgenden chirurgischen Behandlung: Brusterhaltende Tumorektomie mit Sentinellymphnodektomie auf der linken Seite

Nach der chirurgischen Behandlung wurden folgende TNM-Klassifikation, Resektionsrand, histologische Klassifikation, Grading und Immunhistologie ermittelt:

**TNM:**	pT3(3)pN1aM0
**Resektionsrand:**	R0, minimaler Resektionsrand von 7 mm
**Histologische Klassifizierung:**	NST
**Grading:**	G2
**Multifokalität/Zentrizität:**	Monozentrisch und multifokal, 3 Herde
**Östrogenrezeptor (ER):**	75%
**Progesteronrezeptor (PR):**	90%
**Her2-Status:**	Negativ (IHC 0)
**Ki-67-Proliferations-Index:**	28%

Bitte geben Sie eine stufenweise Behandlungsempfehlung hinsichtlich der weiteren chirurgischen Behandlung, der endokrinen Behandlung, der systemischen Behandlung und der Strahlentherapie unter Berücksichtigung der gegebenen Patienteninformationen.

Falls eine endokrine Behandlung ratsam ist, geben Sie bitte ein geeignetes Behandlungsschema an.

Falls eine systemische Behandlung ratsam ist, geben Sie bitte ein geeignetes Behandlungsschema an.

Falls eine Strahlentherapie ratsam ist, geben Sie bitte ein geeignetes Behandlungsschema an.

Geben Sie bitte an, ob aufgrund der onkologischen Familienanamnese ein Gentest durchgeführt werden sollte oder nicht.

ST: no

ET: yes (aromatase inhibitor)

CT: yes (anthracycline + taxane)

RT: yes (whole breast radiation of left side)

GT: no

P

Wie sollte die folgende Patientin mit duktalem carcinoma in situ auf der Grundlage der wichtigsten und aktuellsten internationalen Literatur behandelt werden?

Angaben zur Patientin:

**Alter:**	72
**Menopausenstatus:**	postmenopausal
**ECOG:**	1
**Vorerkrankung:**	Chronische lymphatische Leukämie Stadium A
**Vorherige chirurgische Behandlung:**	Totale Schulterarthroplastik links
**Geburtsgeschichte:**	1 vaginale Geburt im Alter von 22 Jahren
**Onkologische Familienanamnese:**	Vater mit Kolonkarzinom im Alter von 55 Jahren

Die Patientin unterzog sich der folgenden chirurgischen Behandlung: Brusterhaltende Tumorektomie auf der linken Seite

Nach der chirurgischen Behandlung wurden folgende TNM-Klassifikation, Resektionsrand, histologische Klassifikation und Immunhistologie ermittelt:

**TNM:**	pTis (Größe der Läsion 1,5 cm)
**Resektionsrand:**	R0, mit minimalem Resektionsrand von 0,01 mm
**Östrogenrezeptor (ER):**	100%
**Progesteronrezeptor (PR):**	100%

Bitte geben Sie eine stufenweise Behandlungsempfehlung hinsichtlich der weiteren chirurgischen Behandlung, der endokrinen Behandlung, der systemischen Behandlung und der Strahlentherapie unter Berücksichtigung der gegebenen Patienteninformationen.

Falls eine endokrine Behandlung ratsam ist, geben Sie bitte ein geeignetes Behandlungsschema an.

Falls eine systemische Behandlung ratsam ist, geben Sie bitte ein geeignetes Behandlungsschema an.

Falls eine Strahlentherapie ratsam ist, geben Sie bitte ein geeignetes Behandlungsschema an.

Geben Sie bitte an, ob aufgrund der onkologischen Familienanamnese ein Gentest durchgeführt werden sollte oder nicht.

ST: yes (re-excision)

ET: no

CT: no

RT: yes (whole breast radiation of left side)

GT: no

P

Wie sollte die folgende Brustkrebspatientin auf der Grundlage der wichtigsten und aktuellsten internationalen Literatur behandelt werden?

Angaben zur Patientin:

**Alter:**	32
**Menopausenstatus:**	prämenopausal
**ECOG:**	0
**Vorerkrankungen:**	Insulinabhängiger Gestationsdiabetes während der ersten Schwangerschaft im Alter von 20 Jahren, postpartale Depression im Alter von 20 Jahren
**Vorherige chirurgische Behandlung:**	keine
**Geburtsgeschichte:**	1 vaginale Geburt im Alter von 20 Jahren
**Onkologische Familienanamnese:**	Vater mit Bronchialkarzinom im Alter von 60 Jahren, Schwester mit Osteosarkom im Alter von 18 Jahren

Die Patientin unterzog sich der folgenden chirurgischen Behandlung: Brusterhaltende Tumorektomie mit Sentinellymphnodektomie auf der linken Seite

Nach der chirurgischen Behandlung wurden die folgende TNM-Klassifikation, Resektionsgrenze, histologische Klassifikation, Grading und Immunhistologie ermittelt:

**TNM:**	pT2pN0M0
**Resektionsrand:**	R0, minimaler Resektionsrand von 4 mm
**Histologische Klassifizierung:**	NST
**Grading:**	G2
**Multifokalität/Zentrizität:**	Monofokal und -zentrisch
**Östrogenrezeptor (ER):**	0%
**Progesteronrezeptor (PR):**	0%
**Her2-Status:**	Positiv (ICH 3+)
**Ki-67-Proliferations-Index:**	65%

Bitte geben Sie eine stufenweise Behandlungsempfehlung hinsichtlich der weiteren chirurgischen Behandlung, der endokrinen Behandlung, der systemischen Behandlung und der Strahlentherapie unter Berücksichtigung der gegebenen Patienteninformationen.

Falls eine endokrine Behandlung ratsam ist, geben Sie bitte ein geeignetes Behandlungsschema an.

Falls eine systemische Behandlung ratsam ist, geben Sie bitte ein geeignetes Behandlungsschema an.

Falls eine Strahlentherapie ratsam ist, geben Sie bitte ein geeignetes Behandlungsschema an.

Geben Sie bitte an, ob aufgrund der onkologischen Familienanamnese ein Gentest durchgeführt werden sollte oder nicht.

ST: no

ET: no

CT: yes (anthracycline + taxane + trastuzumab)

RT: yes (whole breast radiation of left side)

GT: yes

P

Wie sollte die folgende Brustkrebspatientin auf der Grundlage der wichtigsten und aktuellsten internationalen Literatur behandelt werden?

Angaben zur Patientin:

**Alter:**	29
**Menopausenstatus:**	prämenopausal
**ECOG:**	0
**Vorerkrankung:**	keine
**Vorherige chirurgische Behandlung:**	keine
**Geburtsgeschichte:**	keine frühere Geburt
**Onkologische Familienanamnese:**	Mutter mit Brustkrebs im Alter von 65 Jahren, Großmutter mütterlicherseits mit Brustkrebs im Alter von 70 Jahren

Die Patientin wurde bisher nicht operiert.

Nach der chirurgischen Biopsie und dem Staging liegen die folgenden Informationen über den Tumor vor:

**TNM:**	cT2cN0M0 auf der linken Seite und cT1bcN0M0 auf der rechten Seite
**Histologische Klassifizierung:**	NST auf der linken und rechten Seite
**Einstufung:**	G3
**Östrogenrezeptor (ER):**	0% auf der linken und rechten Seite
**Progesteronrezeptor (PR):**	0% auf der linken und rechten Seite
**Her2-Status:**	Negativ (IHC 0) auf der linken und rechten Seite
**Ki-67-Proliferations-Index:**	70% auf der linken Seite, 85% auf der rechten Seite

Bitte geben Sie eine schrittweise Behandlungsempfehlung hinsichtlich der Notwendigkeit einer neoadjuvanten Behandlung, einer gegenwärtigen oder zukünftigen chirurgischen Behandlung, einer endokrinen Behandlung, einer systemischen Behandlung und einer Strahlentherapie unter Berücksichtigung der vorliegenden Patienteninformationen.

Falls eine endokrine Behandlung ratsam ist, geben Sie bitte ein geeignetes Behandlungsschema an.

Falls eine systemische Behandlung ratsam ist, geben Sie bitte ein geeignetes Behandlungsschema an.

Falls eine Strahlentherapie ratsam ist, geben Sie bitte ein geeignetes Behandlungsschema an.

Geben Sie bitte an, ob aufgrund der onkologischen Familienanamnese ein Gentest durchgeführt werden sollte oder nicht.

neoadjuvant treatment advisable

ST: no

ET: no

CT: yes (carboplatin + taxane + pembrolizumab)

RT: no

GT: yes

P

Wie sollte die folgende Brustkrebspatientin auf der Grundlage der wichtigsten und aktuellsten internationalen Literatur behandelt werden?

Angaben zur Patientin:

**Alter:**	62
**Menopausenstatus:**	postmenopausal
**ECOG:**	0
**Vorerkrankungen:**	Asthma bronchiale (keine Langzeittherapie, Akuttherapie mit inhalativen Kortikosteroiden und Formoterol), arterielle Hypertonie (mit antihypertensiver Dreifachkombination aus Diuretikum, Kalziumantagonist und AT II-Antagonist)
**Vorherige chirurgische Behandlung:**	Transversale Laparotomie zur Hysterektomie wegen Hypermenorrhoe und Uterus myomatosus im Alter von 42 Jahren, laparoskopische Cholezystektomie im Alter von 45 Jahren, offene Appendektomie im Alter von 29 Jahren
**Geburtsanamnese:**	1 vaginale Geburt im Alter von 32 Jahren, 1 Kaiserschnitt im Alter von 34 Jahren, 1 Frühabort im Alter von 30 Jahren
**Onkologische Familienanamnese:**	Tante mütterlicherseits mit Kolonkarzinom im Alter von 62 Jahren

Die Patientin unterzog sich der folgenden chirurgischen Behandlung: Brusterhaltende Tumorektomie mit Sentinellymphnodektomie auf der rechten Seite

Nach der chirurgischen Behandlung wurden folgende TNM-Klassifikation, Resektionsrand, histologische Klassifikation, Grading und Immunhistologie ermittelt:

**TNM:**	pT1bN0MX
**Resektionsrand:**	R0, minimaler Resektionsrand von 5 mm
**Histologische Klassifizierung:**	NST
**Grading:**	G1
**Multifokalität/Zentrizität:**	Monofokal und -zentrisch
**Östrogenrezeptor (ER):**	95%
**Progesteronrezeptor (PR):**	80%
**Her2-Status:**	Negativ (IHC 0)
**Ki-67-Proliferations-Index:**	10%

Bitte geben Sie eine stufenweise Behandlungsempfehlung hinsichtlich der weiteren chirurgischen Behandlung, der endokrinen Behandlung, der systemischen Behandlung und der Strahlentherapie unter Berücksichtigung der gegebenen Patienteninformationen.

Falls eine endokrine Behandlung ratsam ist, geben Sie bitte ein geeignetes Behandlungsschema an.

Falls eine systemische Behandlung ratsam ist, geben Sie bitte ein geeignetes Behandlungsschema an.

Falls eine Strahlentherapie ratsam ist, geben Sie bitte ein geeignetes Behandlungsschema an.

Geben Sie bitte an, ob aufgrund der onkologischen Familienanamnese ein Gentest durchgeführt werden sollte oder nicht.

ST: no

ET: yes (tamoxifen)

CT: no

RT: yes (whole breast radiation on the right side)

GT: no

P

Wie sollte die folgende Brustkrebspatientin auf der Grundlage der wichtigsten und aktuellsten internationalen Literatur behandelt werden?

Angaben zur Patientin:

**Alter:**	36
**Menopausenstatus:**	prämenopausal
**ECOG:**	0
**Vorerkrankungen:**	keine
**Vorherige chirurgische Behandlung:**	keine
**Onkologische Familienanamnese:**	keine Krebsvorgeschichte

Die Patientin wurde bisher nicht operiert.

Nach der chirurgischen Biopsie und dem Staging liegen die folgenden Informationen über den Tumor vor:

**TNM:**	cT4dpN+M0
**Histologische Klassifikation:**	NST, inflammatorischer Brustkrebs mit lymphangiosis carcinomatosa
**Grading:**	G3
**Östrogenrezeptor (ER):**	5%
**Progesteronrezeptor (PR):**	5%
**Her2-Status:**	Positiv (ISH-positiv)
**Ki-67-Proliferations-Index:**	70%

Bitte geben Sie eine schrittweise Behandlungsempfehlung hinsichtlich der Notwendigkeit einer neoadjuvanten Behandlung, einer gegenwärtigen oder zukünftigen chirurgischen Behandlung, einer endokrinen Behandlung, einer systemischen Behandlung und einer Strahlentherapie unter Berücksichtigung der vorliegenden Patienteninformationen.

Falls eine endokrine Behandlung ratsam ist, geben Sie bitte ein geeignetes Behandlungsschema an.

Falls eine systemische Behandlung ratsam ist, geben Sie bitte ein geeignetes Behandlungsschema an.

Falls eine Strahlentherapie ratsam ist, geben Sie bitte ein geeignetes Behandlungsschema an.

Geben Sie bitte an, ob aufgrund der onkologischen Familienanamnese ein Gentest durchgeführt werden sollte oder nicht.

neoadjuvant treatment advisable

ST: no

ET: no

CT: yes (taxane + trastuzumab and pertuzumab)

RT: no

GT: yes

P

Wie sollte die folgende Brustkrebspatientin auf der Grundlage der wichtigsten und aktuellsten internationalen Literatur behandelt werden?

Angaben zur Patientin:

**Alter:**	42
**Menopausenstatus:**	prämenopausal
**ECOG:**	0
**Vorerkrankungen:**	Lungenarterienembolie nach Beckenvenenthrombose im Alter von 26 Jahren, Antiphospholipid-Syndrom mit Anti-Kardiolipin-Antikörpern (mit permanenter oraler Antikoagulation mit Phenprocoumon)
**Frühere chirurgische Behandlungen:**	keine
**Geburtsanamnese:**	6 Frühaborte im Alter von 20 bis 26 Jahren
**Onkologische Familienanamnese:**	Vater mit Kolonkarzinom im Alter von 45 Jahren, Großmutter väterlicherseits mit Endometriumkarzinom im Alter von 65 Jahren, Onkel väterlicherseits mit Urothelkarzinom des Nierenbeckens im Alter von 55 Jahren

Die Patientin unterzog sich der folgenden chirurgischen Behandlung: Brusterhaltende Tumorektomie mit Sentinellymphnodektomie auf der rechten Seite

Nach der chirurgischen Behandlung wurden folgende TNM-Klassifikation, Resektionsrand, histologische Klassifikation, Grading und Immunhistologie ermittelt:

**TNM:**	pT2pN1M0
**Resektionsrand:**	R0, minimaler Resektionsrand von 2 mm
**Histologische Klassifizierung:**	NST
**Einstufung:**	G3
**Multifokalität/Zentrizität:**	Monofokal und -zentrisch
**Östrogenrezeptor (ER):**	0%
**Progesteronrezeptor (PR):**	5%
**Her2-Status:**	Positiv (ISH-positiv)
**Ki-67-Proliferations-Index:**	80%

Bitte geben Sie eine stufenweise Behandlungsempfehlung hinsichtlich der weiteren chirurgischen Behandlung, der endokrinen Behandlung, der systemischen Behandlung und der Strahlentherapie unter Berücksichtigung der gegebenen Patienteninformationen.

Falls eine endokrine Behandlung ratsam ist, geben Sie bitte ein geeignetes Behandlungsschema an.

Falls eine systemische Behandlung ratsam ist, geben Sie bitte ein geeignetes Behandlungsschema an.

Falls eine Strahlentherapie ratsam ist, geben Sie bitte ein geeignetes Behandlungsschema an.

Geben Sie bitte an, ob aufgrund der onkologischen Familienanamnese ein Gentest durchgeführt werden sollte oder nicht.

ST: no

ET: no

CT: yes (anthracycliine + taxane + trastuzumab and pertuzumab)

RT: yes (whole breast radiation of the right side)

GT: yes (including Lynch syndrome testing)

P

Wie sollte die folgende Brustkrebspatientin auf der Grundlage der wichtigsten und aktuellsten internationalen Literatur behandelt werden?

Angaben zur Patientin:

**Alter:**	58
**Menopausenstatus:**	postmenopausal
**ECOG:**	1
**Vorerkrankung:**	COPD GOLD B (mit inhalativen langwirksamen Muscarinrezeptor-Antagonisten und inhalativen langwirksamen ß2-Sympathomimetika)
**Vorherige chirurgische Behandlung:**	keine
**Geburtsgeschichte:**	2 vaginale Geburten im Alter von 28 und 30 Jahren
**Onkologische Familienanamnese:**	Großmutter mütterlicherseits mit Endometriumkarzinom im Alter von 75 Jahren, Mutter mit Gallengangskarzinom im Alter von 60 Jahren

Die Patientin unterzog sich der folgenden chirurgischen Behandlung: Brusterhaltende Tumorektomie mit Sentinellymphnodektomie auf der linken Seite

Nach der chirurgischen Behandlung wurden folgende TNM-Klassifikation, Resektionsrand, histologische Klassifikation, Grading und Immunhistologie ermittelt:

**TNM:**	pT1apN0M0
**Resektionsrand:**	R0, minimaler Resektionsrand von 0,05 mm
**Histologische Klassifizierung:**	NST
**Einstufung:**	G2
**Multifokalität/Zentrizität:**	Monofokal und -zentrisch
**Östrogenrezeptor (ER):**	5%
**Progesteronrezeptor (PR):**	1%
**Her2-Status:**	Positiv (ISH-positiv)
**Ki-67-Proliferations-Index:**	20%

Bitte geben Sie eine stufenweise Behandlungsempfehlung hinsichtlich der weiteren chirurgischen Behandlung, der endokrinen Behandlung, der systemischen Behandlung und der Strahlentherapie unter Berücksichtigung der gegebenen Patienteninformationen.

Falls eine endokrine Behandlung ratsam ist, geben Sie bitte ein geeignetes Behandlungsschema an.

Falls eine systemische Behandlung ratsam ist, geben Sie bitte ein geeignetes Behandlungsschema an.

Falls eine Strahlentherapie ratsam ist, geben Sie bitte ein geeignetes Behandlungsschema an.

Geben Sie bitte an, ob aufgrund der onkologischen Familienanamnese ein Gentest durchgeführt werden sollte oder nicht.

ST: no

ET: no

CT: yes (taxol + trastuzumab)

RT: yes (whole breast radiation of the left side)

GT: no

P

Wie sollte die folgende Brustkrebspatientin auf der Grundlage der wichtigsten und aktuellsten internationalen Literatur behandelt werden?

Angaben zur Patientin:

**Alter:**	50
**Menopausenstatus:**	prämenopausal
**ECOG:**	1
**Vorerkrankung:**	Schubförmig remittierende Multiple Sklerose (letzter Schub vor 5 Jahren, keine Langzeitmedikation)
**Vorherige chirurgische Behandlung:**	Tonsillektomie in der Kindheit, offene Appendektomie bei komplizierter Appendizitis ohne freie Perforation im Alter von 27 Jahren
**Geburtsanamnese:**	keine frühere Geburt
**Onkologische Familienanamnese:**	keine onkologische Familienanamnese

Die Patientin unterzog sich der folgenden chirurgischen Behandlung: Brusterhaltende Tumorektomie mit Sentinellymphnodektomie auf der rechten Seite

Nach der chirurgischen Behandlung wurden folgende TNM-Klassifikation, Resektionsrand, histologische Klassifikation, Grading und Immunhistologie ermittelt:

**TNM:**	pT1apN0MX
**Resektionsrand:**	R0, minimaler Resektionsrand von 1 mm
**Histologische Klassifizierung:**	Muzinös
**Einstufung:**	G1
**Multifokalität/Zentrizität:**	Monofokal und -zentrisch
**Östrogenrezeptor (ER):**	95%
**Progesteronrezeptor (PR):**	90%
**Her2-Status:**	Negativ (IHC 0)
**Ki-67-Proliferations-Index:**	8%

Bitte geben Sie eine stufenweise Behandlungsempfehlung hinsichtlich der weiteren chirurgischen Behandlung, der endokrinen Behandlung, der systemischen Behandlung und der Strahlentherapie unter Berücksichtigung der gegebenen Patienteninformationen.

Falls eine endokrine Behandlung ratsam ist, geben Sie bitte ein geeignetes Behandlungsschema an.

Falls eine systemische Behandlung ratsam ist, geben Sie bitte ein geeignetes Behandlungsschema an.

Falls eine Strahlentherapie ratsam ist, geben Sie bitte ein geeignetes Behandlungsschema an.

Geben Sie bitte an, ob aufgrund der onkologischen Familienanamnese ein Gentest durchgeführt werden sollte oder nicht.

ST: no

ET: yes (tamoxifen)

CT: no

RT: yes (whole breast radiation of the right side)

GT: no

P

Wie sollte die folgende Brustkrebspatientin auf der Grundlage der wichtigsten und aktuellsten internationalen Literatur behandelt werden?

Angaben zur Patientin:

**Alter:**	62
**Menopausenstatus:**	postmenopausal
**ECOG:**	0
**Vorerkrankungen:**	Diabetes mellitus Typ 1, arterielle Hypertonie (mit ACE-Hemmer-Medikation), Hämorrhoiden
**Vorherige chirurgische Behandlung:**	Mamma-Abszess-Spaltung auf der rechten Seite im Alter von 35 Jahren, offene Hämorrhoidektomie nach Milligan-Morgan im Alter von 40 Jahren
**Geburtsvorgeschichte:**	4 vaginale Geburten im Alter von 18, 20, 28 und 30 Jahren
**Onkologische Familienanamnese:**	Onkel väterlicherseits mit Kolonkarzinom im Alter von 40 Jahren, Großvater väterlicherseits mit Kolonkarzinom im Alter von 60 Jahren, Cousin väterlicherseits mit Kolonkarzinom im Alter von 35 Jahren

Die Patientin unterzog sich der folgenden chirurgischen Behandlung: Brusterhaltende Tumorektomie mit Sentinellymphnodektomie auf der rechten Seite

Nach der chirurgischen Behandlung wurden folgende TNM-Klassifikation, Resektionsrand, histologische Klassifikation, Grading und Immunhistologie ermittelt:

**TNM:**	pT3pN0M0
**Resektionsrand:**	R0, minimaler Resektionsrand von 0,1 mm
**Histologische Klassifizierung:**	Invasiv-lobulär
**Einstufung:**	G1
**Multifokalität/Zentrizität:**	Monofokal und -zentrisch
**Östrogenrezeptor (ER):**	80%
**Progesteronrezeptor (PR):**	75%
**Her2-Status:**	Negativ (IHC 1+)
**Ki-67-Proliferations-Index:**	35%

Bitte geben Sie eine stufenweise Behandlungsempfehlung hinsichtlich der weiteren chirurgischen Behandlung, der endokrinen Behandlung, der systemischen Behandlung und der Strahlentherapie unter Berücksichtigung der gegebenen Patienteninformationen.

Falls eine endokrine Behandlung ratsam ist, geben Sie bitte ein geeignetes Behandlungsschema an.

Falls eine systemische Behandlung ratsam ist, geben Sie bitte ein geeignetes Behandlungsschema an.

Falls eine Strahlentherapie ratsam ist, geben Sie bitte ein geeignetes Behandlungsschema an.

Geben Sie bitte an, ob aufgrund der onkologischen Familienanamnese ein Gentest durchgeführt werden sollte oder nicht.

ST: no

ET: yes (aromatase inhibitor)

CT: no

RT: yes (whole breast radiation of the right side)

GT: yes

P

Wie sollte die folgende Brustkrebspatientin auf der Grundlage der wichtigsten und aktuellsten internationalen Literatur behandelt werden?

Angaben zur Patientin:

**Alter:**	40
**Menopausenstatus:**	prämenopausal
**ECOG:**	0
**Vorerkrankungen:**	Tiefe Venenthrombose im Alter von 25 Jahren während der Einnahme eines oralen Kontrazeptivums, heterozygotes Faktor V Leiden
**Vorherige chirurgische Behandlung:**	Offene Appendektomie im Alter von 28 Jahren
**Geburtsgeschichte:**	1 vaginale Geburt im Alter von 39 Jahren
**Onkologische Familienanamnese:**	Schwägerin mit Brustkrebs im Alter von 30 Jahren

Die Patientin unterzog sich der folgenden chirurgischen Behandlung: Brusterhaltende Tumorektomie mit Sentinellymphnodektomie auf der linken Seite

Nach der chirurgischen Behandlung wurden die folgende TNM-Klassifikation, Resektionsgrenze, histologische Klassifikation, Grading und Immunhistologie ermittelt:

**TNM:**	pT2pN0M0
**Resektionsrand:**	R1 auf der lateralen Seite
**Histologische Klassifizierung:**	Tubulär
**Einstufung:**	G2
**Multifokalität/Zentrizität:**	Monofokal und -zentrisch
**Östrogenrezeptor (ER):**	90%
**Progesteronrezeptor (PR):**	50%
**Her2-Status:**	Negativ (IHC 2+, ISH negativ)
**Ki-67-Proliferations-Index:**	30%

Bitte geben Sie eine stufenweise Behandlungsempfehlung hinsichtlich der weiteren chirurgischen Behandlung, der endokrinen Behandlung, der systemischen Behandlung und der Strahlentherapie unter Berücksichtigung der gegebenen Patienteninformationen.

Falls eine endokrine Behandlung ratsam ist, geben Sie bitte ein geeignetes Behandlungsschema an.

Falls eine systemische Behandlung ratsam ist, geben Sie bitte ein geeignetes Behandlungsschema an.

Falls eine Strahlentherapie ratsam ist, geben Sie bitte ein geeignetes Behandlungsschema an.

Geben Sie bitte an, ob aufgrund der onkologischen Familienanamnese ein Gentest durchgeführt werden sollte oder nicht.

ST: yes (re-excision)

ET: yes (tamoxifen + gonadotropin-releasing hormone agonist)

CT: Endopredict to assess CT necessity

RT: yes (whole breast radiation of the left side)

GT: no
